# Diet Item Details: Reporting Checklist for Feeding Studies Measuring the Dietary Metabolome (DID-METAB Checklist)—Explanation and Elaboration Report on the Development of the Checklist by the DID-METAB Delphi Working Group

**DOI:** 10.1016/j.advnut.2025.100420

**Published:** 2025-04-14

**Authors:** Jessica JA Ferguson, Erin D Clarke, Jordan Stanford, María Gómez-Martín, Tammie Jakstas, Clare E Collins, DID-METAB Delphi Working Group, Kathryn L Beck, Clare E Collins, Catalina Cuparencu, David P De Souza, Kerith Duncanson, Mar Garcia-Aloy, Lihi Godny, James O Hill, Elaine Holmes, Deborah A Kerr, Rachel Kimble, Francine Z Marques, Megan A McCrory, Charlotte E Mills, George Moschonis, Kay Nguo, Dorit Samocha-Bonet, Matthew Snelson, Maria H Traka, Caroline J Tuck

**Affiliations:** 1 School of Health Sciences, College of Health Medicine and Wellbeing, The University of Newcastle, Callaghan, New South Wales, Australia; 2 Food and Nutrition Research Program, Hunter Medical Research Institute, New Castle, New South Wales, Australia

**Keywords:** metabolomics, dietary assessment, feeding study, metabolome, biomarkers, nutrition, dietary patterns, personalized nutrition

## Abstract

Metabolomics is a postgenomic, systems-based discipline offering valuable applications in nutrition research, including the use of objective biomarkers to characterize dietary intake and metabolic responses more accurately. A scoping review identified the need for reporting guidance on dietary information in the form of a checklist to ensure reproducibility of human feeding studies that are measuring the diet-related metabolome. In this study, we aimed to gain consensus on a core outcome set pertaining to diet-related item details (DIDs) and recommendations for reporting DIDs to inform development of a reporting checklist. The goal of this checklist is to guide researchers on the minimum level of content and detail required for reporting dietary information in human feeding studies measuring the metabolome. A 2-stage online Delphi process encompassing 5 survey rounds with international experts in clinical trial design, feeding study intervention implementation, metabolomics, and/or human biospecimen analyses was conducted. A core outcome set encompassing 29 DIDs and accompanying recommendations was developed across 5 domains: dietary intervention—modeling (8 DIDs), dietary intervention—implementation (3 DIDs), dietary assessment (9 DIDs), adherence and compliance monitoring (4 DIDs), and bias (5 DIDs). The reporting guideline (DID-METAB Checklist) was generated and accepted by the international expert working group in the final survey round. All experts agreed that relevant journals should include the checklist as a suggested reporting tool for relevant studies and/or used alongside existing reporting tools. This report provides examples, explanations and elaboration for each recommendation including examples from published literature and references. The DID-METAB Checklist will be a key tool to advance the standardized reporting for feeding studies assessing the metabolome. Implementation of this tool will enable the ability to better interpret data and ensure global utility of results for furthering the advancement of metabolomics in nutrition research and future precision and personalized nutrition strategies.

**Statement of significance**

This work provides a novel reporting guideline for improving the comparability and reproducibility of controlled human feeding studies examining the nutritional metabolome. This guideline includes a reporting checklist for use by authors when publishing dietary intervention information from human feeding studies examining the metabolome.

## Introduction

Nutritional metabolomics is a relatively recent field that attempts to identify exogenous metabolites, levels of bioactive compounds from dietary intake, and the impact of diets on endogenous metabolism, including metabolites derived from the gut microbiome [1]. Suboptimal diet and nutritional imbalance are one of the leading risks for the global burden of noncommunicable diseases [2]. The need to improve dietary patterns remains a global issue, however, understanding of diet–disease relationships are limited due to the reliance on self-reported data, with only few validated biomarkers of dietary intake [3]. Precision and personalized nutrition strategies offer promise for targeted, tailored, and efficacious dietary interventions that accommodate human variability [4]. The application of metabolomics in the field of precision and personalized nutrition is emerging, offering an approach to address limitations of self-reported dietary assessment methods that possess inherent challenges such as measurement error, validation of data collection tools, and recall bias by providing an objective measurement of dietary components or downstream metabolites [[5], [6], [7]]. Identifying exogenous and endogenous metabolites is likely to play a key role in the development and implementation of personalized nutrition interventions. Studies have shown that dietary intake improves to a greater extent in individuals who received personalized nutrition advice compared with that in those with generalized dietary advice [8,9]. Specifically, the pan-European Food4Me RCT demonstrated that compared with generalized dietary advice, targeted personalized nutrition advice based on dietary intake, phenotype, and genotype was more effective for improving food group, nutrient intakes, and overall dietary patterns [10,11]. For future translation of precision nutrition strategies into practice, the evidence base of metabolomics requires further standardization to facilitate replication using standardized methodologies and metabolite quantification procedures, as well as a consistent constructs for reporting the methodologies and study findings.

Human dietary feeding studies using metabolomics as an objective dietary assessment method in response to nutrition interventions may involve single food, partial, or whole diet provision and/or dietary prescription [12]. However, dietary intervention methods, including administration of the diet/food provision, dietary assessment, compliance monitoring, and other aspects vary, as does the level of detail used to report this information. This variation makes it challenging to compare results and synthesize the evidence base. To address this gap, we conducted a scoping review to synthesize the methodologic components of controlled human feeding studies designed to measure the metabolome in biospecimens, focusing on plasma, serum, and urine following dietary interventions [13]. The review identified that several studies lacked sufficient detail when reporting dietary intervention information. Extensive variability was noted in methods used in controlled human feeding studies, particularly regarding specific characteristics of dietary patterns administered, methods of implementing dietary feeding interventions, and tools and procedures used to record and assess dietary intake. This issue is not inherent only to the field of nutritional metabolomics, with quality of reporting of dietary interventions previously demonstrated as unsatisfactory [[14], [15], [16], [17]]. To improve future comparability and reproducibility of controlled human feeding studies examining the metabolome, it is important to publish detailed information using standardized approaches within the protocol about dietary interventions being tested, including information about included or restricted foods, food groups, and meal plans provided. Strategies to control for individual variability, such as crossover study design with an adequate washout period, statistical adjustment methods, dietary-controlled run-in periods, or provision of standardized meals or test foods throughout the study should also be considered.

## Aim and Scope

The primary aim was to gain consensus among topic experts on a core outcome set (COS) pertaining to diet-related item details (DIDs), as well as standard reporting recommendations of DIDs in human feeding studies measuring the metabolome. This effort is intended to assist with future research protocol design and facilitate consistency and standardization within and across research studies. The secondary aim (informed by the primary aim) was to develop a reporting guideline (DID-METAB Checklist) for use by researchers conducting human feeding study interventions measuring the metabolome and by journal reviewers and editors to standardize the reporting of core DIDs. Encouraging researchers to use the DID-METAB Checklist during study conceptualization and design, development of feeding interventions, and when preparing manuscripts for publishing the findings will help strengthen methodologic rigor and consistency of the research methods, dissemination of findings, and ultimately, translation to personalized nutrition in practice. The purpose of this elaboration and explanation report was to detail the approach used to gain expert consensus on a COS, drawing on similar efforts and to outline the methodology used in developing the DID-METAB Checklist. This report provides examples, explanations, and elaboration for each item of the checklist including examples from published literature and references.

## Methods

The approach for developing this elaboration and explanation report was based on the guidelines for developers of health research reporting guidelines [18] and was modeled off similar efforts [[19], [20], [21]]. The precision and personalized nutrition (PPN) team (JJAF, EDC, JS, MG-M, TJ, CEC) have collective expertise in human clinical and experimental research design, conduct and implementation of human feeding interventions, dietary assessment methodology, human biospecimen collection and analysis, and design and management of Delphi processes. Development of the COS using the Delphi process was conducted in accordance with the Core Outcome Set-Standards for Development: The COS-STAD recommendations [22]. Delphi techniques are used across disciplines to develop an expert-based judgment about an epistemic question [23]. Although techniques vary, Delphi’s are generally structured group communication processes whereby issues where knowledge is incomplete or uncertain are evaluated by subject matter experts using an iterative process [24,25]. In health sciences, this is often aimed at finding a consensus [26]. This study was approved by the University of Newcastle’s Human Research Ethics Committee (H-2023-0405). Development of the reporting guideline has been registered on the Enhancing the Quality and Transparency of Health Research (EQUATOR) Network database in the clinical trials section (Available at: https://www.equator-network.org/library/reporting-guidelines-under-development/reporting-guidelines-under-development-for-clinical-trials/#METAB; 30 July 2024).

### Identification of core DIDs and reporting set, that is, the COS

A list of items to be reported relating to the design, implementation, assessment, and monitoring of dietary interventions used in human feeding studies (referred to as DIDs) was identified from a systematic scoping review previously published by the research team [13]. The list was further examined by the PPN team with DIDs/examples added, phrasing refined, and DIDs categorized into domains. Key learnings from the PPN team’s design and conduct of a human feeding study investigating the diet-related metabolome in response to a healthy compared with that in response to a typical Australian diet [27,28] also informed this process. A final list of 28 DIDs, including examples of DIDs, were identified and categorized under 5 domains: *1*) dietary intervention—modeling, *2*) dietary intervention—implementation, *3*) dietary assessment, *4*) adherence and compliance monitoring, and *5*) bias.

### Recruitment

Selection of the expert panel is outlined in Text Box 1. There is no published agreement on the optimal size of an expert group/panel. A map summarizing 12 systematic reviews of Delphi techniques undertaken across health sciences identified that the number of included experts varied [23]. Therefore, a pragmatic approach was used to ensure that a range of opinions are also garnered to adequately represent the field. At a minimum, we aimed to recruit 15 experts with 5 across each area of expertise at completion of the 2-stage Delphi (i.e., total completers of both stage 1 and stage 2). Since it has been reported previously that completion rates for Delphi questionnaires are 80% or greater [23,[29], [30], [31]], the goal was to recruit an additional 12 experts to account for potential nonresponse. Hence, expressions of interest were screened until 30 eligible experts were identified or the recruitment period had lapsed, whichever occurred first.

TEXT BOX 1 **SELECTION OF EXPERT PANEL**

A list of international experts was identified that included researchers with experience and expertise in clinical and experimental trial design with expertise in administering dietary feeding interventions in humans (highly prescriptive and/or partial/total diet provision); design and conduct of human feeding studies with the primary purpose to profile the diet-related metabolome and/or experts in nutritional metabolomics more broadly; and specific diet-related biospecimen analyses and interpretation (faecal microbial metabolites, plasma, and urine metabolites) and/or another related field. Experts were identified from key publications examining the metabolome following dietary interventions or metabolomics more broadly; key speakers at relevant conferences, researchers with relevant expertise identified through the research team’s existing networks, and directors of metabolomics laboratories, and research groups with expertise in conducting human feeding studies. Experts across career stages and backgrounds internationally were invited (e.g., nutrition and dietetic researchers, biochemists, faecal microbial scientists, and human metabolism scientists). Researchers with limited expertise (e.g., undergraduate students), higher degree researchers (e.g., Masters or PhD students), and researchers unable to write/read English language were excluded.

Alt-text: TEXT BOX 1

During the 8-wk recruitment period (mid-December 2023 to mid-February 2024), 67 experts were identified and emailed a generic invitation letter, which included the participant information statement and a URL to the online self-administered eligibility form. The eligibility form included information about the study, collected basic demographic information, and concluded with the ability to provide consent if the experts were deemed eligible, at which point study enrolment occurred. In addition, recruitment of experts was advertised through the PPN team’s independent social media accounts (e.g., X and LinkedIn) and invited experts were also encouraged to share the invitation with colleagues, which assisted with word-of-mouth recruitment. The experts’ identities remained unknown to others in the expert group.

### Delphi survey

A 2-staged Delphi methodology with 5 survey rounds was used to gain consensus on a COS of DIDs. Stage 1, consisting of 2 rounds, focused on identifying the core DIDs, although stage 2, comprising 3 rounds, addressed the associated priority and level of reporting recommendations for the final list of DIDs. The Delphi approach used self-administered online surveys with individual feedback from experts used to reach a consensus. The Delphi was conducted between mid-February 2024 and mid-July 2024, Figure 1 summarizes the Delphi process.

**FIGURE 1:**
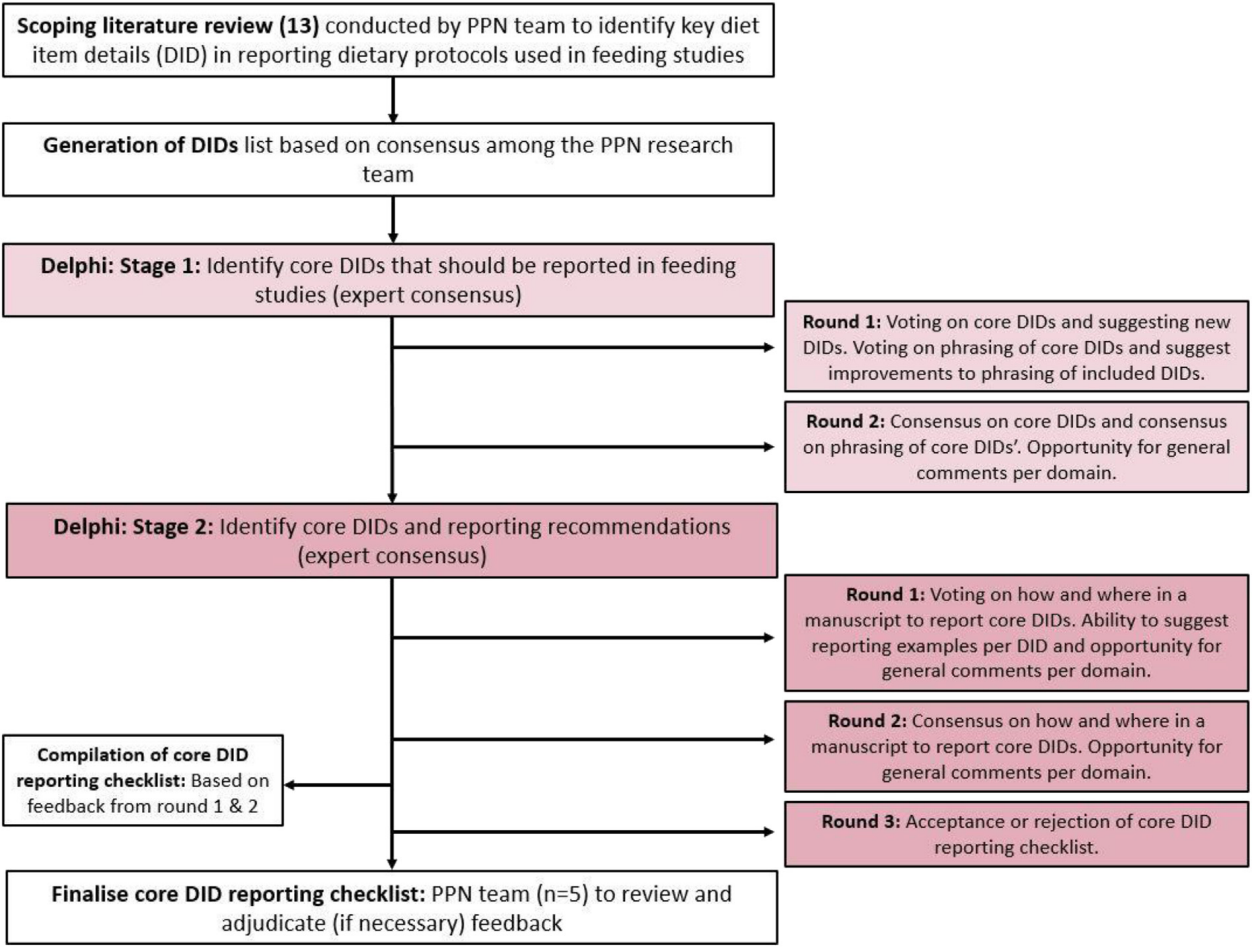
The Delphi process. DID, diet item detail; PPN, Precision and Personalized Nutrition.

At the start of each round (survey), a general summary was provided including the updated DIDs list and their corresponding examples to enter that respective round. For the last round of stage 2, a brief introduction regarding how the checklist is intended to be implemented was also provided.

Experts were given between 2 and 3 wk to complete each survey, with a 2- to 3-wk break in between, and 3 scheduled reminders sent during this period to participants with incomplete responses only. Experts who did not complete a survey round were ineligible to continue to the next round.

All surveys including the initial screening eligibility and recruitment form were inputted into QuestionPro Survey Software, an online system capable of hosting surveys, collating, and securely storing data. After each round, results were extracted into an Excel spreadsheet and grouped thematically to inform consensus (JJAF). Recurring themes were identified, categorized, and coded in duplicate by a second investigator from the PPN team (TJ) for a random 10% sample of DIDs each round, where applicable to ensure consistency. Additional email reminders were needed to ensure technical issues did not prevent a participant from being able to access the survey links. To achieve consensus, a cut-off agreement rate of 70% was used to decide on DID inclusion/exclusion and reporting recommendations for binary questions. Detailed description of the methodology used for each round within each stage of the Delphi including consensus generation is provided in Supplementary Methods.

## Results

### Expert characteristics

Of the 67 experts that were invited to participate, 30 were recruited, with 1 ineligible due to <1 y of expertise in eligible research area. Therefore, 29 experts were invited to participate in the first Delphi round. Four did not respond, thus 25 commenced stage 1 round 1, with 3 lost-to-follow up after stage 1 round 2. Data obtained from the 3 experts lost-to-follow up remained in the study. Twenty-two (88%) completed all 5 rounds (Supplemental Figure 1). Over half resided in Australia, with the majority female (82%) and aged between 35 and 44 y (40%), followed by 55–64 y (27%). All except 1 expert reported having >1 area of expertise specific to this study and in total, the expert pool contributes >200 y of research expertise in relevant fields. Over 90% had expertise in clinical and experimental design of dietary interventions and human feeding studies (partial/whole diet provision) (Supplemental Table 1).

### Consensus on the core DIDs (stage 1)

In stage 1 round 1, all DIDs reached a consensus rating for inclusion of ≥70%, except for 3 (DID5, DID8, and DID13), with 2 DIDs (DID11 and DID27) on the lower end (76% and 72% consensus, respectively) (Supplemental Table 2). The phrasing of all DIDs was agreed upon as indicated by ≥70% consensus except for 2 (DID1 and DID16) and phrasing for DID8 was borderline (70.6%). Ten DIDs received consensus ratings between 70% and 80% (DID3, DID4, DID7, DID8, DID12, DID14, DID17, DID18, DID27, and DID28). Although DID13 did not reach consensus for inclusion (68%), consensus for phrasing was high (94.1%). Considering this along with expert commentary and discussion among the PPN team, DID13 was merged with DID12 to form 1 DID (now DID12). The majority of rephrasing was minor to enhance consistency and clarity in the interpretation of the DIDs. Four DIDs remained unchanged in their phrasing for entry into round 2.

A total of 29 DIDs entered round 2. Although DID5 and DID8 did not reach consensus for inclusion in round 1 (<70%, but > 50%), the phrasing of these DIDs did meet consensus. Therefore, the PPN team discussed and aligned on minor rephrasing to improve clarity, and DID5 and DID8 achieved consensus in round 2 (92% and 76%, respectively), with the rest achieving a consensus rating of 80%–100%. At the end of round 2, recurring expert commentary relating to the terminology of DIDs was captured, pertaining to consistency required in relation to the DIDs either being phrased as items (as originally intended) or instructions/command. After discussion, the PPN team agreed to keep the terminology as items, therefore minor rephrasing of DIDs removed action words such as “…should be reported” or “…discuss in detail.” Twenty-three DIDs remained unchanged for entry into stage 2.

In stage 1 round 1, several experts used the free-text fields as an opportunity to provide suggestions for additional examples to be listed for specific DIDs and/or amendments to be made for existing DID examples. Some also commented that the examples were helpful when interpreting DIDs. Therefore, in stage 1 round 2, the PPN team included an open-ended question to invite experts to list any further examples they believed should be included for a specific DID. Although the examples do not form part of the core DID set nor are they intended as an exhaustive list, the PPN team decided that the examples would be a part of the DID reporting checklist to assist with usability by authors in the field.

### Consensus on the location of reporting and level of detail of reporting of core DIDs and their reporting sets (stage 2)

Following stage 2 round 1, all DIDs were rated as a recommendation for some level of reporting in the methods section of research articles, using criterion outlined in Supplemental Table 2. Nearly all DIDs (24/29) were recommended to be reported with a brief description in the methods section; the rest were recommended to provide a detailed description in the methods section. Although 16 DIDs were identified as suitable for reporting in a table, this was only recommended for DID18; the reporting recommendations for the remaining DIDs were identified as consider (*n* = 5 DIDs) or were optional (*n* = 10 DIDs). Similarly, 11 DIDs were identified as suitable for reporting in a figure, 10 of these were optional to report in this manner, 1 to consider (DID26), and none were recommended. All DIDs were identified as requiring at a minimum optional reporting in a supplementary file. Seventeen were identified as being recommended to be reported in detail, 11 to consider reporting in detail, and only 1 (DID25) as optional to report at all. Following stage 2 round 2, all DIDs met consensus for reporting in some manner in the methods section of a research article with some degree of detail. Thirty-two reporting sets (i.e., level of detail and location) spanning across 26 DIDs did not meet consensus.

### Reporting checklist (DID-METAB Checklist)

All experts accepted the final DID-METAB Checklist (Table 1) [32]. All experts agreed with the PPN Team’s recommendation that the checklist should be used alongside existing tools (e.g. , as an extension of item 5 in CONSORT 2010 Statement for RCTs, or item 11 in SPIRIT 2013 checklist for protocol items in intervention trials), and that relevant journals should recommend the checklist for relevant studies.

**TABLE 1:** Diet-related item details (DIDs): reporting checklist for feeding studies measuring the human dietary metabolome (DID-METAB checklist).

Details to include when describing the methodology of feeding studies and the appropriate sections for reporting this information
The DID-METAB Checklist is for reporting dietary details used in intervention and control groups in human feeding studies related to the dietary metabolome. The aim is to ensure adequate reporting of dietary methodology and to facilitate replication. Other study components are covered by existing reporting statements and checklists. Further information is included in the DID-METAB guide paper and should be used alongside the DID-METAB Checklist.
Grouped under 5 domains, are 29 diet-related item details (DIDs) with a hierarchy of reporting recommendations. Those labeled as consider or optional are additional suggested recommendations that may guide the methodology choices of study design. Examples of content to report for each DID are also provided in the table.
For each DID reporting recommendation, please specify where it is documented by indicating the manuscript page number, supplementary materials, or other resources (e.g., protocol paper or preprint) in the last (where reported) column. If a DID is not applicable to the intervention or study design, please use NA.
It is strongly recommended that this checklist is used in conjunction with the CONSORT 2010 Statement1, as an extension of Item 5 when a randomized clinical feeding trial is being reported, or in conjunction with the SPIRIT 2013 Statement2 as an extension of Item 11 for clinical feeding trial protocols. DID-METAB Checklist can also be used in conjunction with checklists relevant to other study designs (see www.equator-network.org). Although the DID-METAB Checklist is intended for the methods section of a paper (unless explicitly stated as supplementary file), in some cases, specific items may be more relevant to be reported in other sections, for example, results or discussion.

DID No.	DID	Recommendations for reporting item	Where reported^1^: page no. or supplementary no.
Domain 1—Dietary intervention—modeling			
1	Methods and/or tools used to design the nutritional/dietary characteristics of the dietary intervention(s) and control diet(s) used. • Detailed methods reported to replicate a published position or well-established therapeutic diet or dietary trend such as DASH, Mediterranean Diet, for example, <X mg sodium, X% sat fat (X serves of fruits and vegetables), including references. • Software used including version number, for example, ProNutra ver 1.0	Detailed description (up to ∼250 words)	
		Detailed description for novel or nonstandard method and/or tools and/or if journal is nonnutrition/dietetic in a supplementary file. Provide an example of method/tools in a supplementary file.	
		Optional: describe in a table	
2	References to population-based dietary guidelines, survey data, and/or published therapeutic diets (where possible) that inform the design of dietary interventions. • National or International population–based dietary guidelines • National survey data	Brief description (couple of sentences)	
		Consider: detailed description in a supplementary table.	
3	Method(s) used for personalizing and/or modifying the dietary intervention(s) and control diet(s). This may include implementing dietary substitutions to accommodate specific diet or nutritional needs; individual preferences; anthropometric, biochemical, or clinical profile; and/or product availability/seasonality. • Energy matching dietary intervention by upscaling or downscaling food items according to participant’s basal energy intake or calculated energy requirements • Food/meal substitutes due to food allergies, intolerances, aversions, or specific nutritional requirements	Brief description (couple of sentences)	
		Detailed description in a supplementary table(s), figure(s) and/or provide examples.	
		Optional: describe in a table	
4	a) Food composition database and/or reference material used to analyze the nutritional content of the dietary intervention(s) and control diet(s), including references. • Australian Food Composition Database (e.g., AUSNUT 2013 formerly NUTTAB) • Software programs used including reference to version number, for example, FoodWorks and ProNutra	Brief description (couple of sentences)	
		Consider: detailed description in supplementary table.	
5	b) Details of the applicability of the food composition database and/or reference material to the population being studied. Explanation of how the food composition database is representative of the population being studied, including references. Or, if the database used is not representative of the population, explain why it was used and/or why it was considered the best available or an appropriate substitute.	Brief description (couple of sentences)	
6	Method(s) used to standardize dietary intake within groups. • Food library reference with predetermined food/meal substitutes for each dietary intervention. • Full (or at least partial) provision of foods, meals, and/or raw ingredients. • Where food is supplied, the following may be relevant: grocery order placed by study investigators, meals made in test/commercial kitchen, participants required to consume X number of meals at research facility under supervision, participants to collect foods from research site X times per week, minimal food preparation or cooking required. • Identical meal plans provided to participants • Support resources, for example, foods/meals to choose when eating out and takeaway for each dietary intervention • Description of food form, for example, mashed, pieces, and powder	Detailed description (up to ∼250 words)	
		Detailed description in a supplementary table(s)	
7	Qualitative and quantitative characteristics of all dietary intervention(s) described in a reproducible manner. • Portion sizes, required serves per food group, and food choices/characteristics, for example, β-carotene–rich fruits and vegetables, and wholegrain vs refined grain products • Nutrient targets • Example meal plan or rotating menu • Timing of food intake and food/meal patterns	Detailed description in a separate paragraph under its own subheading	
		Provide example meal plan or rotating menu in a supplementary file	
		Consider: detailed description in table for each diet group	
8	Personnel responsible for designing and developing the dietary intervention(s) and control diet(s), including who developed menu/meal plans; provided dietary education; and any documents/resources provided to the participants clearly identified along with their relevant qualifications. • Research dietitian, registered nutritionist/dietitian, accredited practicing dietitian, and research team member in liaison with any of the aforementioned. • Or list relevant qualifications, certifications, training undertaken, and/or experience for personnel involved.	Brief description (couple of sentences)	
		Detailed description and/or provide documentation of participant resources in supplementary file	
Domain 2—Dietary intervention—implementation			
9	The proportion of food and/or beverages provided for each dietary intervention. • All or full provision of diet should be stated or inferred • Partial or expressed as a % or proportion of total food intake diet, for example, 80% or 90% of all foods and beverages needed for individual consumption were provided to participants • Provision of any key food items relevant to the dietary intervention(s), for example, provision of olive oil for a Mediterranean diet • If relevant, provide specific weight of food(s) provided, for example, 100 g berries • Description of any food allowances, for example, condiments, spices, seasonings, water, and noncaloric beverages	Brief description (couple of sentences)	
		Consider: detailed description in a supplementary table(s) for each diet group and/or examples of participant handouts/resources provided	
10	Nature of the food and/or beverages provided (e.g., recipe of test food/meal, raw ingredients, cooking instructions, preprepared meals, and combination), storage conditions, and how this was provided to participants (e.g., delivered to their home, fed onsite, and collected from supermarket). • Raw ingredients provided which participants used to assemble/cook own meals; only preprepared/cooked meals provided; combination or raw ingredients; and preprepared meals. • Participants collected grocery order from supermarket or research facility, study food was delivered to participants’ house, or participants were provided with a gift card to purchase groceries, and so on. • Foods prepared by a research test kitchen, third-party quality-controlled kitchen, or commercial kitchen to ensure standardization	Brief description (couple of sentences)	
		Detailed description in a supplementary table(s) and/or figure(s) where applicable and/or examples of participant handouts/resources provided	
11	Contingency strategies to ensure food provision remained as close to the original protocol. Researchers performed quality control checks by placing/confirming grocery orders with participants, keeping food stock on hand of essential menu items for participants to collect if required, use of a predeveloped food library/substitutes food list for out-of-stock items	Brief description (couple of sentences)	
		Detailed description in supplementary file	
Domain 3—Dietary assessment			
12	Dietary assessment method(s) used (strengths, limitations, reliability and validity, including whether it has been validated in the population being studied) or reason(s) why a dietary assessment method was not used. • Stating the name of tools, whether it was validated and in what population including references (where relevant). • Stating if calibrated against weighed food records (e.g., ASA-24 and Intake-24) and/or validated using strategies such as direct observation, an objective measure (e.g., doubly labeled water), and recovery biomarkers. • Stating whether participants were asked to return all uneaten food, whether this was weighed/recorded against food provided, and so on. • Stating whether all food was eaten at research facility under supervision	Brief description (couple of sentences) including statement on validation and relevant references	
13	a) Description of the dietary assessment method(s) used to examine food items recorded (or consumed) and estimate (or quantify) portion size. • Serves of each food group, grams of each food or food group via 24-h recalls, and so on. • If validated, reference the validation paper relating to the method/tool	Brief description (couple of sentences)	
		Detailed description in supplementary file and/or example of method/tool used if applicable	
14	b) Description of the frequency of conducting the dietary assessment method(s), including number of days (if applicable). Serial 24-h recalls 4 times per study period, or two 3-d food records at baseline and postintervention, food frequency questionnaires, weighed food records weekly, and direct meal observation	Described in 1 sentence or very briefly	
15	c) Description of the timing of the dietary assessment method(s) used in relation to the timing of biospecimen data collection. Dietary intake collected 24 h before blood collection or dietary intake collected at time of biospecimen (urine, blood, fecal, and saliva) sample collection	Described in 1 sentence or very briefly	
		Optional: report in a figure	
16	d) Description of how the dietary assessment method(s) were administered and by whom. Interviewer administered (study investigators) or self-administered (e.g., participant via e-form and survey)	Described in 1 sentence or very briefly	
17	e) Description of how the quality and accuracy of the administration of the dietary assessment method(s) was assured. Quality control checks, for example, results reviewed by study investigators and clarified with participant where relevant, and random phone call audits	Described in 1 sentence or very briefly	
18	Qualitative and quantitative dietary intake data for all dietary intervention(s) and control diet(s) and whether data presented are for reported intake or based on foods/beverages provided/prescribed only. • Tabulated servings of foods by food groups for each feeding arm (and whether this is reflective of provision/prescription, reported intake, or both). • Tabulated nutritional information for each feeding arm (and whether this is reflective of provision/prescription, reported intake, or both). • Incorporating deviations to dietary protocol, either incorporated as part of dietary assessment method (for actual intake reporting) or retrofitted/overlaid on dietary protocol (for intake presented as food provided).	Detailed description (up to ∼250 words)	
		Detailed tabulation for each diet group	
		Detailed description in supplementary table(s) and/or figure(s)	
19	Methods used to assess and account for consumption of nonstudy food and/or beverage items, that is, foods that were consumed but not provided or prescribed as part of diet protocol. • Log of nonstudy food/beverage items consumption documented in an online or paper-based pro forma list • Captured in dietary assessment method	Brief description (couple of sentences)	
		Detailed description in supplementary file	
20	Procedure used to match food composition of dietary intervention items provided with actual consumption data, reporting conversion factors or assumptions made (if applicable). • Food composition databases, for example, Australian Food Composition Database (formerly NUTTAB), used to analyze nutrient intake. • Sensitivity analysis to adjust for prescribed vs actual dietary intake	Brief description (couple of sentences)	
Domain 4—Adherence and compliance monitoring			
21	Method(s), tools, and/or resources used to optimize engagement and adherence to diet intervention(s) and whether this was the same for all diet interventions (where applicable). • Energy-matched/tailoring to food preferences (where possible) and how, for example, unit foods • Itemized meal plan with portion sizes • Nonstudy food consumption guide, for example, takeout • Provide a meal box/lunch box to support out-of-home consumption • Meal box reminder cards of what to pack • Check-in phone calls • Variability in repeated menus to prevent fatigue (where applicable to research question) • Rotating menu with cycle length that prevents fatigue, for example, 7 d • Reminders, for example, automated email reminders/texts or phone calls. • Examining satiety (visual analog scale) and/or food acceptability questionnaire • Consultation with research team, for example, email, phone, study interval check-in applications/communication	Brief description (couple of sentences)	
		Detailed description in supplementary table(s), figure(s), and/or include examples	
22	Method(s) used to monitor adherence to dietary intervention(s), stating whether this involved objective methods (e.g., biomarkers or known metabolites) and whether the method(s) used was the same for all dietary interventions (where applicable) and control diet(s). • Use of marker foods with known metabolites that are measured in biospecimen. • Objective measures such as *p*-aminobenzoic acid to examine sample collection completeness • Where biospecimens are used, state type of biospecimen, for example, plasma, urine, and the nature of collection, for example, spot urine, and 24 h collection. • Dietary assessment methods, for example, 24 h recalls, food records/diaries, and direct meal observation. • Weighing of uneaten portions and/or uneaten food (including spilled food) returned or photographed • Specific compliance questionnaire and/or checklist • Full (or at least partial) diet provision • Supporting resources, for example, itemized meal plan, meal box reminders, takeout meal ideas • Check-in phone calls/regular consultation with researchers	Detailed description (up to ∼250 words).	
		Consider: detailed description in supplementary file	
23	How nonadherence and/or outliers were managed. • Consumption of nonprescribed food, nonconsumption of prescribed foods, describe cutoffs that identify nonadherence. • Describe procedures that identified outliers to the dietary protocol, for example excessive metabolite concentrations that cannot be reasonably explained. Include description of cutoffs.	Brief description (couple of sentences)	
		Consider: detailed description in supplementary file	
24	Detailed description of how unforeseen circumstances (e.g., acute illness and personal circumstances) that required deviation or adjustment to dietary protocol were managed (e.g., temporary pause in dietary intervention with recommencement after a suitable washout period, adjustments in nutritional requirements, or rescheduling of clinic appointments). • Temporarily pause feeding intervention periods and/or reschedule clinic appointments with a suitable washout period for recovery of illness • Ceasing dietary intervention followed by suitable washout period before recommencing dietary intervention • Adjustment in nutritional requirements (if relevant)	Brief description (couple of sentences)	
		Detailed description in supplementary file	
Domain 5—Bias			
25	How selection bias in dietary intervention allocation were mitigated or addressed. • Randomized order of dietary intervention (crossover study) or allocation to dietary intervention (parallel study). • Stratified random sampling (individuals stratified for sex and any other characteristics known to influence the dietary metabolome and/or other key outcomes). • If and how blinding was implemented, for example, single and double	Brief description (couple of sentences)	
26	Whether a washout period was used, and if so, what the conditions were, and duration justified. Washout period between dietary interventions such as return to habitual dietary intake or standardized feeding protocol.	Brief description (couple of sentences)	
		Consider: description in a figure	
27	How potential bias in dietary reporting (i.e., misreporting, recall bias, and changing habits as a result of being assessed) were mitigated. • Use of validated dietary assessment methods with visual aids to support accurate recall, for example, ASA-24, Intake-24, and Australian Eating Survey • Use of image-based and/or sensor-based dietary assessment methods • Interviewer-administered dietary assessment methods • Strategies to control for overreporting and underreporting, for example, Goldberg equation [32]	Brief description (couple of sentences)	
		Consider: detailed description in supplementary file	
28	Measures taken to control for potential confounding factors that could influence interindividual and intraindividual variations outside the scope of the study protocol. • Crossover study design so that participants serve as their own controls. • Crossover study design in random order so that there is no order effect. • Provide a standardized dietary run-in phase (e.g., 1–2 wk) before randomization, for example, whole diet feeding, partial diet feeding, and highly prescriptive meal plan. • In a parallel study design, standardized test meals or foods administered at various time points throughout the study. These meals/foods would be provided before concurrently testing metabolomics or other metabolic measures to evaluate individual responses. • Provision of partial or whole diet to reduce variability in food preparation or cooking practices.	Brief description (couple of sentences)	
29	Acknowledgment of the generalizability of the population being studied. Comment on the generalizability of population being studied.	Described in 1 sentence or very briefly	

1 Provide details of where this information is available/sourced from if it is not provided in the current article. For example, citations for published articles or protocol papers, website URL, and/or catalog or report citations. Describe any derivations or deviations from original protocol. We strongly recommend using this checklist in conjunction with the DID-METAB Explanation and Elaboration Report (add citation and DOI), which provides further information.

## How to Use This Report?

This report contains examples and explanations designed to support researchers in using the DID-METAB Checklist items and recommendations when reporting details of study methods. The following section showcases how each DID can be achieved using exemplar excerpts from published studies including explanations of each item’s importance, with further supporting evidence where relevant. This information is not provided for items listed as consider or optional, nor those recommended for supplementary file. For some DIDs, 2 examples have been provided where food was consumed under surveillance situations rather than partially/fully provided and participants consume at home. This study has been registered on the Core Outcome Measures in Effectiveness Trials (COMET Initiative) database (https://www.comet-initiative.org/Studies/Details/3292). The development of the checklist was registered on the EQUATOR Network (https://www.equator-network.org/library/reporting-guidelines-under-development/reporting-guidelines-under-development-for-clinical-trials/#METAB). A copy of the final checklist is available at https://australianeatingsurvey.com.au/did-metab-statement,the EQUATOR Network, and the end of this article and in the DID-METAB COS statement published in *European Journal of Clinical Investigation*.

## Checklist Items

### Domain 1—dietary intervention—modeling

#### DID1: Methods and/or tools used to design the nutritional/dietary characteristics of the dietary intervention(s) and control diet(s) used

Recommendation: Detailed description (up to ∼250 words).

Example:

The Healthy Australian Diet provides foods to adequately meet the recommended servings of the 5 core food groups according to the current Australian Dietary Guidelines for adults [33]. The diet will also aim to meet the acceptable macronutrient distribution ranges and some emphasis will be given to specific nutrient targets such as fiber, added sugars and sodium. For fruits and vegetables, those rich in β-carotene (e.g., carrots, pumpkin, tomatoes, red capsicum and sweet potato) will be emphasized during this dietary pattern. The inclusion of β-carotene–rich foods in this dietary pattern serves to create a clear distinction from the comparator dietary pattern (Typical Australian Diet) in terms of carotenoid intake.
This distinction is particularly relevant for analyzing plasma samples and, more importantly, for detecting differences in measured skin carotenoid levels. Characteristics of the food choices will reflect a high diet quality consistent with recommendations for the 5 core food groups in the Australian Dietary Guidelines. The Typical Australian Diet is based on the most recent data on the nutritional profile of Australians [34], from the Apparent Consumption of Australians report, which is the amount of food and nonalcoholic beverages purchased from food and retail sectors, for example, major supermarkets, smaller outlets, delis, fresh food markets, and butchers, from July 2020 to June 2021 [34]. Fruits and vegetables that are low in β-carotene (e.g., white potato, onion, cauliflower, and pears) will be emphasized during this dietary pattern. Characteristics of the food choices will reflect a poor diet quality consistent with the Apparent Consumption of Foods Among Australians in 2020–2021 [34] (Table 1). [27], p. 3

Explanation: Providing details of the methods/tools used enables researchers to compare and reproduce dietary interventions for their own metabolomics applications. Methods/tools may also include published positions, well-established therapeutic diets, or dietary trends, for example, the Mediterranean diet. Details for software used to assist with the design of dietary interventions could also be provided here. When describing novel or nonstandard methods, tools, and/or if the journal is not nutrition based, this information could be provided in a supplementary file, along with further detailed examples of the methods/tools used.

#### DID2: References to population-based dietary guidelines, survey data, and/or published therapeutic diets (where possible) that inform the design of dietary interventions

Recommendation: Brief description (couple of sentences).

Example:

The Healthy Australian Diet provides foods to adequately meet the recommended servings of the 5 core food groups according to the current Australian Dietary Guidelines for adults [33]…. The Typical Australian Diet is based on the most recent data on the nutritional profile of Australians [34], from the Apparent Consumption of Australians report, which is the amount of food and nonalcoholic beverages purchased from food and retail sectors, for example, major supermarkets, smaller outlets, delis, fresh food markets, and butchers, from July 2020 to June 2021 [34]. [27] p. 3

Explanation: Appropriately referencing the source(s) of key dietary design features enhances reproducibility and enables examination of how to translate findings to other populations. Reference to dietary intake data from national surveys or other published therapeutic diets, functional foods/supplements, and dietary guidelines/recommendations could also be included.

#### DID3: Method(s) used for personalizing and/or modifying the dietary intervention(s) and control diet(s). This may include implementing dietary substitutions to accommodate specific diet or nutritional needs; individual preferences; anthropometric, biochemical, or clinical profile; and/or product availability/seasonality

Recommendation: Brief description (couple of sentences).

Example:

Study diet energy needs were established on the basis of self-reported 4-d food record (4DFR) energy intake together with standard energy estimating equations [35] and data from previous Women’s Health Initiative (WHI) calibration equations [36,37] that include a woman’s BMI, race-ethnicity, and age. For women whose food record energy intake results were less than the correction value [111 of 153 (73%) of the women], food prescriptions were increased proportionally to reach the correction energy value. On average (±SD), an additional 335 ± 220 kcal/d were added. For those with food record results greater than the correction value (∼27% of the women), calories were not changed, because we wanted to ensure that we were providing sufficient food to discourage women from supplementing their controlled diets with nonstudy foods. [38], p. 468.

Explanation: There will be instances where study volunteers have an allergy, intolerance, or aversion to a specific food and/or have nutritional requirements that do not preclude them from participating in the study but require deviations to the original dietary intervention characteristics. These instances should also be included under this item. Additionally, some studies use a single food item to upscale caloric provision to meet individual energy requirements such as a 100-kcal muffin [39] or 100-kcal cookie [40]. Although only a brief description is recommended in the manuscript, a detailed description which may be presented in a table or figure and/or with specific examples as a supplementary file(s) is also recommended.

#### DID4: a) Food composition database and/or reference material used to analyze the nutritional content of the dietary intervention(s) and control diet(s), including references

Recommendation: Brief description (couple of sentences).

Example:

The diets were designed and analyzed using ProNutra software (version 3.4; Viocare) with nutrient values derived from the USDA National Nutrient Database for Standard Reference, Release 26 and the USDA Food and Nutrient Database for Dietary Studies, 4.0. [41], p. e2.

Explanation: Provision of the food composition database and/or references to the tools used enables the reader to examine the translatability of the dietary data with respect to the type and/or recency of database information. It also enhances more accurate reproducibility as future researchers can use the same databases or reference material where applicable. Authors should reference any software used.

#### DID5: b) Details of the applicability of the food composition database and/or reference material to the population being studied

Recommendation: Brief description (couple of sentences).

Example:

The ASA24 uses the AUSNUT 2011–2013 food composition database [42] and quantified intakes of ≤65 nutrients for each ASA24 conducted…. The AES [Australian Eating Survey] uses the AUSNUT 2011–2013 food composition database to calculate nutrient intakes [42]. Diet quality will be assessed using the Australian Recommended Food Score (ARFS), which uses a subset of 70 questions from the AES…. Both the AES and ARFS have been validated in Australian populations from the age of 2 y and is based on 15 y of research [43,44]. [27], p. 6.

Explanation: Reporting the food composition database and/or references to the tools used, aids comparison with future studies aiming to conduct research on the same population. Use of food databases not specific to the reference population will have some limitations such as reduced generalizability, and this should be explained. Explanation regarding decisions about databases/reference materials that are not representative of the population will assist with the interpretation of findings and/or highlight the potential need for a relevant food composition database and/or reference material specific to the population being studied.

#### DID6: Method(s) used to standardize dietary intake within groups

Recommendation: Detailed description (up to ∼250 words).

Example 1 (food provided):

The DGA [Dietary Guidelines for Americans] and typical American diets were prepared at the Beltsville Human Nutrition Research Center research kitchen… The Korean menu items were prepared in bulk by a local Korean chef at her own establishment and under the supervision of the study’s Korean coinvestigators. The Korean diet included 5 food groups: grains (mix of whole and refined); meat, fish, eggs, and beans; vegetables; fruits; and milk and dairy products. The Korean foods were prepared from ingredients using the traditional Korean preparation techniques. All meals were weighed and apportioned to the nearest gram by trained staff at the Beltsville Human Nutrition Research Center kitchen. Menus were developed to provide 2000 kcal/d for a 7-d cycle, and all diets were then scaled by weight to provide an energy intake range of 1800 to 3600 kcal/d to maintain body weight. Menus were developed using the ESHA dietary analysis program (ESHA Food Processor SQL 2011, version 10.8.0; ESHA Research). The nutrient composition of each diet was analyzed in composite aliquots of all menus by an accredited laboratory (Medallion Labs). [45], p. 1085.

Example 2 (food consumed under surveillance):

Participants attended the CRF [clinical research facility] for a 72-h inpatient period on 4 occasions, separated by ≥5 d (appendix p 10). We chose 3 d (72 h) for the inpatient period because most food-derived metabolites are absorbed and eliminated in urine within 48 h, as evidenced in numerous studies (including other studies done in our laboratories) of the kinetics of absorption, bioavailability, and elimination of several food metabolites contributing to the urinary metabolome [46]…. Participants were asked to consume all the food provided and were allowed to drink water as they wished. The expectation to consume all food provided and not to leave the CRF during each visit was fully explained to potential participants before they provided consent to take part in the study. This adherence was monitored strictly: all food was weighed immediately before being given to the participants, and any uneaten food was weighed. [47], p. 186.

Explanation: Authors should describe all methods used to standardize dietary intake within intervention and control groups to guide conduct of future feeding interventions that have similar feeding design features. Detailed description in a supplementary table(s) is also recommended which may include copies of supporting resources provided to participants used to facilitate standardization of dietary intake assessment, for example, guides for eating food away from home; checklist for allowed nonstudy food that is to be supplied by participants; or reference materials used by researchers to retain standardized dietary intake in the event of food/meal substitutes are required.

#### DID7: Qualitative and quantitative characteristics of all dietary intervention(s) described in a reproducible manner

Recommendation: Detailed description in a separate paragraph under its own subheading.

Example:

Prescribed dietary interventions
All foods and beverages were prepared and provided to participants to consume ab libitum by the metabolic kitchen at the NIH Clinical Center. The presented UPF-DP (ultraprocessed food dietary pattern) and UN-DP were matched for total calories, macronutrient composition (∼47% carbohydrate, 36% fat, and 17% protein), total sugars, fiber, and sodium and are described in detail previously [41]. The foods and beverages were classified according to the Nova system [48]. Nova classifies foods and beverages into 4 groups according to the degree of processing [49]. Group 1 includes unprocessed or minimally processed foods, such as fresh, dry, or frozen fruits or vegetables, grains, legumes, meat, fish, and milk, which have undergone minimal processing techniques, such as grinding, cooking, or pasteurization. Group 2 includes processed culinary ingredients, such as table sugar, oils, fats, salt, and other substances that have been extracted, pressed, or centrifuged from foods used for culinary preparation. Group 3 includes processed foods, which include group 1 foods that have culinary ingredients from group 2 added, such as canned fruits, artisanal bread, cheese, or smoked meat. Group 4 includes ultraprocessed foods, which are foods with group 2 ingredients as well as additives not used in culinary preparations, such as flavors, colors, nonnutritive sweeteners, emulsifiers, and other substances, used to increase palatability and sensorial properties of the food or beverage. The UPF-DP was composed of 5% of total EI (%en) from minimally processed foods (Nova group 1), <1 %en culinary ingredients (Nova group 2), 14 %en processed foods (Nova group 3), and 81 %en UPF (Nova group 4). The UN-DP was composed of 88%en minimally processed foods (Nova group 1), 12%en culinary ingredients (Nova group 2), 0%en processed foods (Nova group 3), and 0%en UPF (Nova group 4). Presented meals were identical in composition and amount for each participant [41] and were consumed ad libitum. Pictures and descriptions, including brand names, of all meals and snacks provided to participants can be found here in the Supplementary Materials of the original article [41]. All recipes are available upon reasonable request. [50], p. 2183.

Explanation: Detailed description of qualitative and quantitative characteristics of all dietary intervention arms including any food restrictions. This will enhance reproducibility of interventions and contribute to future synthesis, for example, systematic reviews and meta-analyses. Where the control intervention is standard care, this should also be described in detail and referenced where appropriate. The information provided should be sufficient for the reader to know exactly how to administer the intervention(s) if they were to replicate in their own study. It is recommended that an example meal plan or rotating menu is provided in a supplementary file (Supplemental Table 3).

#### DID8: Personnel responsible for designing and developing the dietary intervention(s) and control diet(s), including who developed menu/meal plans; provided dietary education; and any documents/resources provided to the participants clearly identified along with their relevant qualifications

Recommendation: Brief description (couple of sentences).

Example:

The study dietitian reviewed the 4-d food record (4DFR) and conducted a standardized, in-depth interview to assess usual food choices and patterns that may not have been captured on the 4DFR. Questions included food likes, dislikes, brands, meal patterns, recipes, snacks, and alcohol use; this additional information was used to design the individual planned diets for the feeding study. Food records were entered into the Nutrition Data System for Research (NDS-R; Nutrition Coordinating Center, version 2010; University of Minnesota) software by trained technicians for nutrient analysis and menu planning. [38], p. 468.

Explanation: Ideally personnel with adequate expertise (e.g., dietitian or nutritionist) should lead the design and development of the dietary interventions used in feeding studies, and this should be documented to clarify whether sufficient skillset and surveillance has been implemented. When this is not the case, authors are encouraged to report why. It is recommended that authors provide a detailed description and/or samples of participant resources such as menus, meal plans, or recipes in a supplementary file.

### Domain 2—dietary intervention—implementation

#### DID9: The proportion of food and/or beverages provided for each dietary intervention

Recommendation: Brief description (couple of sentences).

Example:

The entirety of the intervention diets (i.e., 3 main meals and snacks per day) are provided to participants. Participants will provide their own tea, coffee, and fluids and will be instructed to record their intake of all beverages and any nonstudy foods during each dietary intervention phase using the Easy Diet Diary application (Xyris Pty Ltd). [27], p. 4.

Explanation: Description of any food allowances should also be documented, for example, spices, seasonings, water, condiments, and nonalcoholic beverages. Where dietary interventions involved partial food provision, authors should also report what food(s) and beverages were not provided in the intervention, and how these were sourced, for example, purchased by participants with/without instructions. Clarity on the level of food provision is important and may assist with explaining any potential discrepancies or variances observed within and across intervention groups.

#### DID10: Nature of the food and/or beverages provided (e.g., recipe of test food/meal, raw ingredients, cooking instructions, preprepared meals, and combination), storage conditions, and how this was provided to participants (e.g., delivered to their home, fed onsite, and collected from supermarket)

Recommendation: Brief description (couple of sentences).

Example 1 (food provided):

All meals and snacks will be sourced from a supermarket chain and assembled by the research team specifically for the study and participants will collect the food from the nominated supermarket chain closest to their residence. [27], p. 4.

Example 2 (food consumed under surveillance):

Meals were delivered to participants’ rooms 3 times/d, and participants were given 60 min to consume each meal. Participants were instructed to eat as little or as much food as desired. A variety of snacks and bottled water were provided each morning that could be consumed throughout the day ad libitum. [50], p. 2183.

Explanation: It is important to report the nature of food and/or beverage provision to understand the potential variability that may enter with this design feature (e.g., retailer stock variation and participant error in self-purchasing), whether authors controlled for potential variability and/or how this may be mitigated in future studies. It is also recommended that authors provide a detailed description in a supplementary table(s) and/or figure(s) and/or provide examples of participant handouts/resources, where applicable—see further details provided by McCullough et al. [51] in their supplementary file 2. For multicenter trials, the nature of food and/or beverage provision should also be detailed for each study center (Supplemental Table 4).

#### DID11: Contingency strategies to ensure food provision remained as close to the original protocol

Recommendation: Brief description (couple of sentences).

Example 1:

The amounts of foods in the basal diet were also adjusted to accommodate the added F&V [fruit and vegetables], such that both diets provided a similar percent energy from carbohydrate (56%), protein (16%), and fat (28%). All prepackaged foods were purchased in case lots and fresh foods were purchased from the same vendor. [52], p. 891–892.

Example 2:

All food was sourced from the same supermarket chain, with items ordered by the research team and collected by participants from their nearest nominated store. Accredited practicing dietitians selected appropriate substitutes for any out-of-stock items to align as closely as possible with the nutrient content and food group serving targets of the respective dietary intervention. Any substitutions were noted for each participant and communicated accordingly.

Explanation: Information on strategies implemented to account for potential deviations to dietary protocol that are out of direct control of the research team should be described to assist with optimizing future feeding study interventions. Issues such as retailer stock variability, delay or cancellations of grocery orders, and incorrect stock items provided should be prospectively considered by researchers when planning and designing dietary feeding protocols where food is provided to participants for independent consumption (as in example 2 which we have devised). Other strategies may include implementation of quality control checks on grocery orders with participants, keeping essential food stock on hand at the research facility, and having a predeveloped food substitution library for out-of-stock or missing items. It is recommended that further detail on contingency strategies is provided in a supplementary file.

### Domain 3—dietary assessment

#### DID12: Dietary assessment method(s) used (strengths, limitations, reliability, and validity, including whether it has been validated in the population being studied) or reason(s) why a dietary assessment method was not used

Recommendation: Brief description (couple of sentences) including statement on validation and relevant references.

Example:

The Automated Self-Administered 24-h—Australia (ASA24 AUS) Dietary Assessment Tool will be used to assess dietary intake 3 times during each dietary feeding period to quantify intakes during each feeding period and to inform adherence…. The ASA24 is an online, multiple pass, self-administered recall tool reporting information on all food and beverages consumed in the preceding 24 h. The ASA24 uses the AUSNUT 2011–2013 food composition database [42] and quantified intakes of ≤65 nutrients for each ASA24 conducted.
The Australian Eating Survey (AES) is a 135-item self-administered validated semiquantitative FFQ [food frequency questionnaire] that measures usual food and nutrient intakes over the past 3 mo…. The AES uses the AUSNUT 2011–2013 food composition database to calculate nutrient intakes [42]. Diet quality will be assessed using the Australian Recommended Food Score (ARFS)…. Both the AES and ARFS have been validated in Australian populations from the age of 2 y and is based on 15 y of research [43,44]. [27], p. 6

Explanation: Reporting on the validation of the dietary assessment method used is important for the reader to interpret accuracy of the dietary assessment method and hence data on the dietary patterns and/or nutrient intakes of the population being studied, the national recommended dietary guidelines, and/or in accordance with trends in dietary intakes in populations over time [[53], [54], [55]]. It is also recommended that the strengths, limitations, and reliability are described to enable assessment of the suitability of the dietary assessment method for use in future feeding studies. A justification for not using a dietary assessment method is recommended.

#### DID13: a) Description of the dietary assessment method(s) used to examine food items recorded (or consumed) and estimate (or quantify) portion size

Recommendation: Brief description (couple of sentences).

Example 1 (food provided):

Both the Healthy diet and the Control diet were isocaloric based on the evaluation of the habitual diet (calculated from a 4-d food record) during the run-in period. National nutrient databases were used to calculate energy, macronutrient, cholesterol, fiber and micronutrient contents [Aivo Finland Ltd, Turku, based on the database of the National Institute of Health and Welfare, Finland, Dietist XP Software Package, version 3.1 (2009) linked to Swedish Food Database 2009, Sweden, Master Dietist System version 1.235 (2007) based on Danish National Food administration database, Denmark, and The Icelandic Food Composition Database (ISGEM), Iceland]. [56], p. 55

Example 2 (food consumed under surveillance):

At each mealtime, remaining food and beverages were reweighed by study staff, and nutrient and metabolizable EIs were calculated via ProNutra software version 3.4 (Viocare) using the USDA National Nutrient Database for Standard Reference, Release 26, and the USDA Food and Nutrient Database for Dietary Studies, 4.0. [50], p. 2183.

Explanation: Where study foods/beverages are not consumed under surveillance, additional details for how dietary intake was assessed relating to servings of each food group and/or grams of each food group or food item may also be described, for example, using a 24-h recall, dietitian-administered diet history, or other methods. It is recommended that a detailed description and/or example of method/tool used is provided in a supplementary file if applicable.

#### DID14: b) Description of the frequency of conducting the dietary assessment method(s), including number of days (if applicable)

Recommendation: Described in 1 sentence or very briefly.

Example:

In addition to the 4-d food record during the run-in period, the participants kept 4-d food records, as well as at weeks 2, 11 and 17, or 23, before the next visit to the study center for calculations of the dietary intake during the intervention. [56], p. 56

Explanation: Description of the frequency of dietary assessment and breadth of dietary intake captured (e.g., 24 h and 3 d) should be described for all dietary interventions. This provides clarity around the degree of dietary information available in relation to metabolite data, as well as provides insight into dietary protocol adherence measures. In studies where meals are provided and consumed under surveillance, authors should describe whether meal intake was observed by researchers for the entire duration of mealtimes and/or how frequently participants attended research facilities for food/meal consumption.

#### DID15: c) Description of the timing of the dietary assessment method(s) used in relation to the timing of biospecimen data collection

Recommendation: Described in 1 sentence or very briefly.

Example:

The Automated Self-Administered 24-h—Australia (ASA24 AUS) Dietary Assessment Tool will be used to assess dietary intake 3 times during each dietary feeding period to quantify intakes during each feeding period and to inform adherence. The first recall falls within the first week of each phase, the second and the third recalls fall within the second of the phase aligned with biospecimen collection. [27], p. 6.

Explanation: The ability to determine what timeframe dietary intake is assessed with respect to biospecimen data collection. This information enables understanding of whether crossvalidation of dietary metabolites to inform compliance/adherence is possible and/or whether the biological sampling is providing an objective recall of recent (e.g., 24-h recall) dietary intake and is being complemented by information collected from traditional dietary assessment instruments.

#### DID16: d) Description of how the dietary assessment method(s) were administered and by whom

Recommendation: Described in 1 sentence or very briefly.

Example:

They consumed a hot meal, which was weighed to the nearest gram by the research dieticians…. All the foods were precalculated for macronutrient composition and energy content for each individual participant by the research dieticians. [57], p. 1175

Explanation: Information about whether the dietary assessment method was interviewer administered (e.g., dietitian, nutritionist, and trained personnel) or self-administered (e.g., participant led via e-form and hardcopy survey) may be relevant to describe for studies where food was provided to participants for consumption unsupervised. This information enables understanding of the potential impact of reporting bias and/or whether the appropriate personnel were involved in dietary assessment.

#### DID17: e) Description of how the quality and accuracy of the administration of the dietary assessment method(s) was assured

Recommendation: Described in 1 sentence or very briefly.

Example:

Participants kept logs of time of day and number of pouches consumed during the 7 d of the controlled diet. These records were reviewed with the study dietitian to ensure completeness and accuracy. [58], p. e3

Explanation: Given the inherent error and bias associated with self-reported data [5,59,60], strategies should be implemented to optimize quality and accuracy of self-reported dietary intake. These may include interviewer-administered dietary assessment methods by trained personnel and trained personnel crosschecking self-reported data with participants to ensure data input is optimized.

#### DID18: Qualitative and quantitative dietary intake data for all dietary intervention(s) and control diet(s) and whether data presented are for reported intake or based on foods/beverages provided/prescribed only

Recommendation: Detailed description (up to ∼250 words). Detailed tabulation for each diet group.

Example:

Table 1 displays the nutrient targets for each diet and the average estimated servings per day of foods for the 2100-kcal version of the diets. Table 2 displays a sample, 1-d set of meals. The primary distinguishing feature of the 3 diets is their macronutrient composition. By design, each diet was reduced in saturated fat, cholesterol, and sodium, and rich in fruits, vegetables, fiber, potassium, and other minerals at recommended levels [61].
The carbohydrate diet used in this trial is similar to the DASH [Dietary Approaches to Stop Hypertension] diet, except that the carbohydrate intake of the DASH diet was 55% of kcal compared with 58% of kcal in the carbohydrate diet and the protein intake of the DASH diet was 18% of kcal compared with 15% of kcal in the carbohydrate diet. The protein intake was reduced to 15% of kcal to achieve a 10% of kcal contrast with the protein diet. Approximately two-thirds of the increase in protein from the carbohydrate to the protein diets came from plants (legumes, grains, nuts, and seeds). However, sources of protein were varied and included meat, poultry, egg product substitutes, and dairy products. The protein diet included some soy products, but the amount was low, on average just 7.3 g/d. The unsaturated fat diet emphasized monounsaturated fat. This diet included olive, canola, and safflower oils, as well as a variety of nuts and seeds, to meet its target fatty acid distributions. The type of carbohydrate in each diet was similar, as indicated by the total dietary glycemic index (68 in carbohydrate diet, 71 in the protein diet, and 75 in unsaturated fat diet, relative to the white bread index) [62]. [40], p. 2456–2457.

**Figure:**
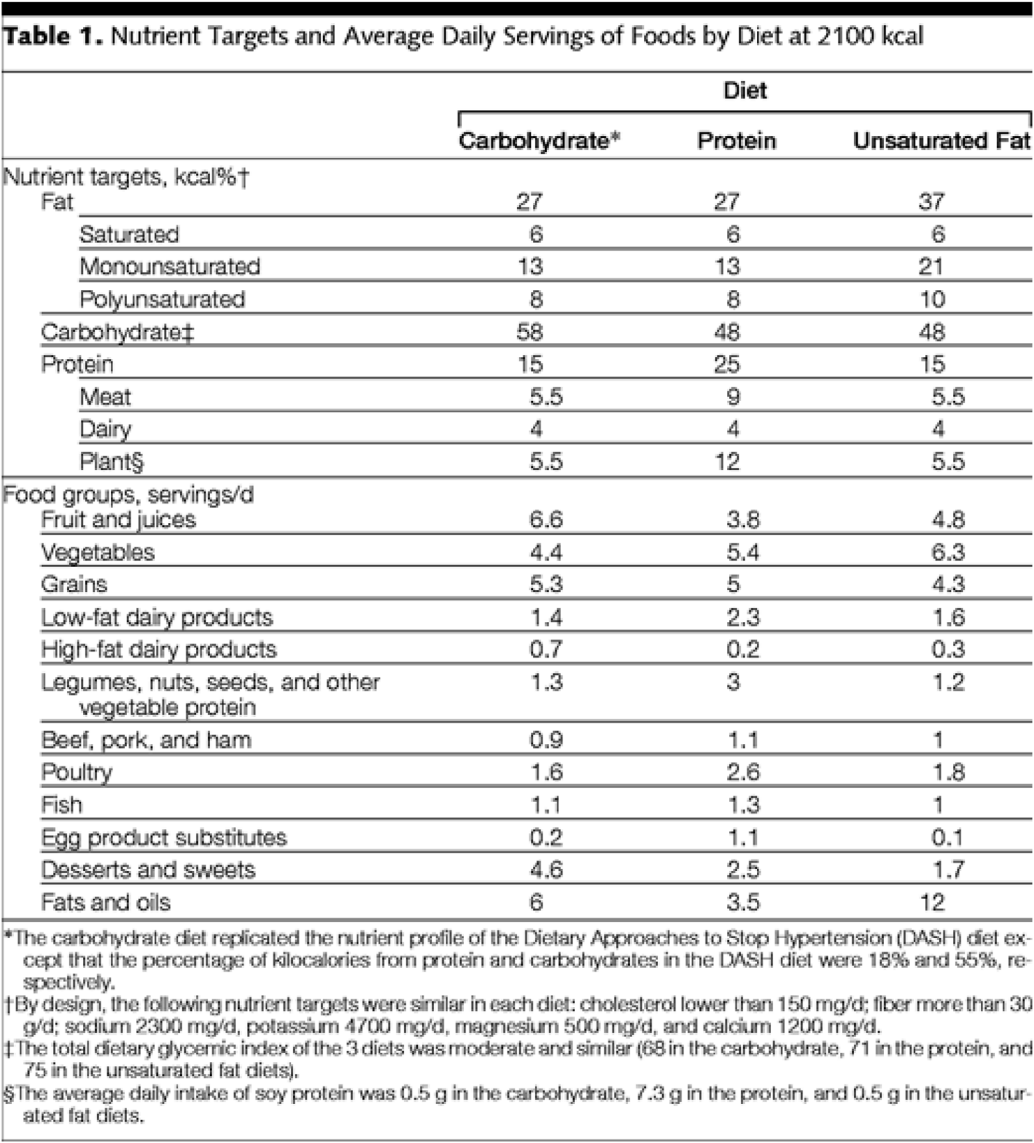

Explanation: Qualitative and quantitative dietary target/intake information facilitates replication, allowing researchers to examine the dietary intervention using food composition databases specific to their population of interest. It should be clear whether data presented represent actual food intake and/or the targeted provision/prescription of food/meals (i.e., the example provided presents the latter). It is recommended that a detailed description is also provided in a supplementary table(s) and/or figure(s).

#### DID19: Methods used to assess and account for consumption of nonstudy food and/or beverage items, that is, foods that were consumed but not provided or prescribed as part of diet protocol

Recommendation: Brief description (couple of sentences).

Example:

Overall compliance with the study diet was assessed using daily food check-off forms; each checklist covered all foods on the study diets and provided space to record any additional nonstudy foods consumed. Participants were encouraged to report any deviations from the study diets and were asked to bring back study foods that were consumed incompletely so that the staff dietitian could weigh the amount of leftover food. [52], p. 892.

Explanation: Consumption of nonstudy food and/or beverage items should be examined and detailed to assist with accounting for potential confounding factors and to optimize interpretation of the dietary metabolome data. Methods used may include a record of nonstudy food/beverages intake using a paper-based pro forma list, online form, diet record application, or an explanation of how this has been captured as part of the dietary assessment method. It is recommended that further details are described in a supplementary file.

#### DID20: Procedure used to match food composition of dietary intervention items provided with actual consumption data, reporting conversion factors or assumptions made (if applicable)

Recommendation: Brief description (couple of sentences).

Example 1:

Food records were entered into the Nutrition Data System for Research (NDS-R; Nutrition Coordinating Center, version 2010; University of Minnesota) software by trained technicians for nutrient analysis and menu planning…. A daily menu checklist, which was used to record consumption of study and nonstudy (when applicable) foods and beverages, also was collected…. Uneaten study foods were returned to the HNL [Human Nutrition Laboratory] (when applicable) and weighed and recorded. Quantities of consumed foods were then reentered into the NDS-R for use in nutrient analysis. [38], p. 468

Example 2:

According to participant self-reports, adherence was high, that is, all study food was consumed and no nonstudy food was eaten on 95% to 96% of person-days on each diet. [40], p. 2459–2460.

**Figure:**
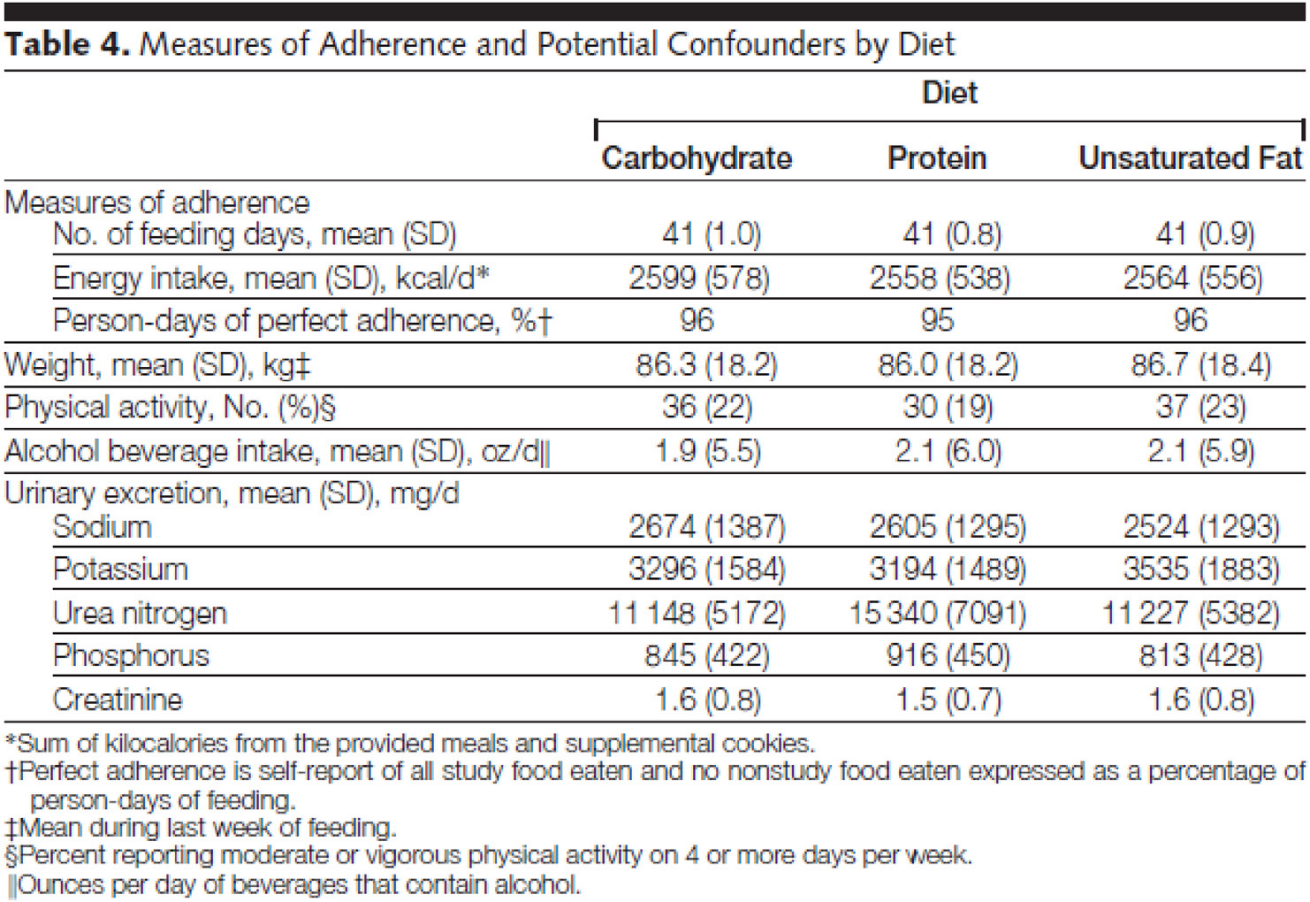

Explanation: Procedures or methods detailing how prescribed/targeted dietary intake compared with actual dietary intake consumed should be addressed to clarify whether results of the dietary metabolome reflect the intended dietary feeding protocol, actual dietary intake, and/or how much dietary intake may have deviated from protocol. This can be achieved by presenting actual dietary intake data (example 1) in addition to qualitative and/or quantitative characteristics of the intended dietary protocol. Where actual dietary intake is not measured or presented, authors should demonstrate that there is no apparent difference between intended dietary intake and actual dietary intake (example 2).

### Domain 4—adherence and compliance monitoring

#### DID21: Method(s), tools, and/or resources used to optimize engagement and adherence to diet intervention(s), and whether this was the same for all diet interventions (where applicable)

Recommendation: Brief description (couple of sentences).

Example:

A study meal box, reminder meal cards, and takeaway/eating out resources respective for each dietary intervention will be provided to participants to assist with consumption of study foods away from the home…. Participants will also undergo 2 short (10–15 min) virtual check-in appointments with study researchers at weeks 3 and 7 via telephone/video call to monitor compliance and adherence to study protocol, monitor adverse events, and have an opportunity to ask any questions. [27], p. 4–5

Explanation: Additional strategies dependent on study aims could include rotating menus with cycle lengths that prevent fatigue, for example, 7 d; automated email/text reminders to eat; monitoring satiety and/or food acceptability using visual analog scales or questionnaires; and conducting nutrition counseling sessions to support adoption of dietary interventions. Tailoring dietary protocols to individual’s basal energy and nutritional requirements will also assist with optimizing adherence and help participants to remain weight stable. Detailed description and/or examples of tools/resources used is also recommended to be provided in a supplementary table(s) or figure(s).

#### DID22: Method(s) used to monitor adherence to dietary intervention(s), stating whether this involved objective methods (e.g., biomarkers or known metabolites) and whether the method(s) used was the same for all dietary interventions (where applicable) and control diet(s)

Recommendation: Detailed description (up to ∼250 words).

Example:

Dietary adherence will be assessed throughout the entire study using: three 24-h recalls per dietary intervention phase, and record keeping of habitual beverage (including alcohol) consumption and food consumed out of the home for example, takeaway, restaurant or café using the Easy Diet Diary application. Presence of plasma metabolites for respective indicator foods for each diet intervention will also be monitored to assess dietary adherence retrospectively. Participants will receive 150 mL orange juice to consume daily at lunchtime in both dietary phases. This food was chosen to monitor compliance and dietary adherence as its metabolite proline betaine is well documented in previous feeding studies [[63], [64], [65], [66]]. 2,5-Dihydroxybenzoic acid, 3,5 dihydroxyphenylpropionic acid [67,68], alkylresorcinols [69,70], and pipecolic acid betaine [71] will signify daily wholegrain bread/ cereal intake during the Healthy Australian Diet, and theobromine will signify daily chocolate intake in the Typical Australian Diet [63,72]. [27], p. 4–5.

Explanation: Several methods may be used to monitor dietary adherence, and this may include use of subjective measures (i.e., self-reported dietary intake), objective measures (i.e., dietary biomarkers/metabolites), or a combination as in the example above. Using food-related biomarkers derived from specific foods as markers of indicator foods or dietary patterns is recommended within controlled feeding studies [73]. Biomarkers of food intake that have been validated, at a minimum, according to their plausibility, robustness, and reliability should be used to monitor compliance/dietary adherence [74]. Validation should include agreement between biomarkers of food intake with traditional or subjective dietary assessment instruments. Additionally, a combination of 2 or more validated food-related biomarkers [[74], [75], [76]] or food biomarker ratios [77,78] should be used to indicate consumption of a food or food group for compliance monitoring purposes as these approaches have been shown to accrue higher specificity and predictability compared with single food-related biomarkers. Metabolomics provides promise for enhancing the reliability of dietary assessment by substituting or complementing subjective dietary instruments with objective markers of food intake.

#### DID23: How nonadherence and/or outliers were managed

Recommendation: Brief description (couple of sentences).

Example:

Compliance was assessed by using a checklist where all products consumed each week were registered. Each food group was ranked equally, and the percentage of intake based on the minimum amounts to be consumed per day was calculated during the whole study period. A person was considered compliant to the protocol if the average of the percentage compliance of all food items eaten during the study was 80%. Only 3 of the subjects were not compliant to the protocol. [79] p. 1386.

Explanation: Description of procedures that identified outliers to the dietary protocol may also be described, such as excessive urinary or plasma metabolite concentrations that cannot be reasonably explained. Where subjective and/or objective measures (e.g., dietary biomarkers) are used, cutoffs determining adherence/nonadherence should be defined along with references (where applicable) to enable replication.

#### DID24: Detailed description of how unforeseen circumstances (e.g., acute illness and personal circumstances) that required deviation or adjustment to dietary protocol were managed (e.g., temporary pause in dietary intervention with recommencement after a suitable washout period, adjustments in nutritional requirements, or rescheduling of clinic appointments)

Recommendation: Brief description (couple of sentences).

Example:

Where individuals experienced a mild acute illness (e.g., flu, common cold, or migraine) not requiring any prescription medication, participants were given the opportunity to cease the intervention phase they were receiving until asymptomatic, from which they then underwent the 2-wk washout period (habitual diet) per protocol, before recommencement of the allocated dietary intervention. All clinic appointments were rescheduled accordingly.

Explanation: Although this was identified as a core item for reporting, it is not adequately reported in the current scientific literature, therefore, we have devised the above example. For future studies, these details may be reported prospectively in the methods section or in the results section of a research article if in relation to circumstances that occurred unexpectedly. It is recommended that a detailed description is provided in a supplementary file as required.

### Domain 5—bias

#### DID25: How selection bias in dietary intervention allocation were mitigated or addressed

Recommendation: Brief description (couple of sentences).

Example:

The randomization list was created by an external statistician (LINK Medical, Norway), using 4 strata—females younger than 50 y, females aged 50 y or older, males younger than 50 y, and males aged 50 y or older—and a block size of 6. The SAS system (R) was used to generate the list. The randomization allocations, selected consecutively, were sent to the food packaging personnel on demand, according to strata information of newly recruited subjects. All food items were packed in boxes outside the study center, and only the people packing the boxes knew who would be allocated to which group. Each box was labeled with an ID number, and the closed boxes with the food items were delivered to the study center. At the study center, the subjects received the ID-labeled boxes. Thus, the study was double blinded as neither the subjects nor the nutritionist who handed out the food boxes knew which group the subjects were assigned to. [79], p. 1385

Explanation: Although randomization and blinding will be addressed in other reporting guidelines such as items 8–11 in the CONSORT checklist [80], characteristics known to influence the dietary metabolome and/or other key outcomes should be described (e.g., tabulated participant characteristics) and/or considered in post hoc statistical analyses where relevant.

#### DID26: Whether a washout period was used, and if so, what the conditions were, and duration justified?

Recommendation: Brief description (couple of sentences).

Example:

A washout period of 2–4 wk separated the feeding periods. During the washout, participants ate their own food. [40], p. 2457.

Explanation: Conditions implemented during the washout period enable assessment as to whether carryover effects were mitigated, as well as optimizing accuracy in replication. In crossover studies where a washout period was not used, a description of why and/or other measures implemented to minimize carryover effects is encouraged to assist the reader to examine the potential impact on data interpretation. Investigating potential carryover bias during statistical analysis, such as mixed-effects models [81], using postinterventional metabolome assessments, or measuring the metabolome before the start of each intervention arm, provides alternative strategies for investigating carryover effects and accounting for them retrospectively. For example: “To partially address the lack of run-in or washout periods, we compared ad libitum energy intake during the final week of each test diet period and the substantial diet differences persisted” [41].

#### DID27: How potential bias in dietary reporting (i.e., misreporting, recall bias, and changing habits as a result of being assessed) were mitigated

Recommendation: Brief description (couple of sentences).

Example:

Along with the food diary, each participant received a validated photography booklet that contained 13 series of colored photographs, each with 4 different portion sizes ranging from small to large. Food amounts were estimated in predefined household units (e.g., glasses, pieces, or tablespoons) or from photographs. We included specific precoded questions about the control/experimental food items used in the intervention, and the participants had to mark when they used the control/experimental food items and how much was eaten. [79], p. 1385–1386

Explanation: Using a validated dietary assessment method with visual aids and/or using weighed food records to support accurate recall are ideal strategies for mitigating potential bias in dietary reporting. Other strategies include image-based or sensor-based methods, interviewer administered dietary reporting tools, or the Goldberg method to categorize misreporters of FFQs [food frequency questionnaires] and 24-h recalls [82]. Consumption of some or the majority of dietary interventions under direct surveillance is a strategy that lowers dietary reporting bias. However, this is not always feasible or practical, and observation of dietary intake is known to impact consumption patterns, notably lowering energy intake [83]. Strategies used to mitigate heightened awareness should be described for such studies.

#### DID28: Measures taken to control for potential confounding factors that could influence interindividual and intraindividual variations outside the scope of the study protocol

Recommendation: Brief description (couple of sentences).

Example 1:

Briefly, 21 adults with BMI (in kg/m^2^) of ≥27, aged 18–40 y, underwent a run-in phase comprising weight monitoring for 4 wk, a hypocaloric diet made entirely from food (not formula) in a metabolic kitchen for 12 wk to achieve weight loss corresponding to 10%–15% of initial body weight, and weight stabilization for 4 wk. During a subsequent test diet phase, participants consumed 3 diets prepared in a metabolic kitchen, each for a 4-wk period, in a random order. To ensure balance and unpredictability, we prepared 30 assignments for order of diet, comprising 5 replicates of the 6 possible orders, grouped in Latin squares with random permutation within and between squares. [84], p. 548

Explanation: Although crossover studies are optimal for minimizing interindividual and intraindividual variation, they may not always be feasible due to greater participant burden, typically longer study duration, and thus subsequent dropouts [85].

Example 2:

All participants consumed the average Danish diet ad libitum in the run-in period, except for the last 3 d when participants were provided specific average Danish diet foods in specific amounts to ensure that they were in energy balance. This standardization of the diet served to standardize participants to the same diet before the first clinical examination at week 0. [86], p. 37

Explanation: In parallel feeding studies, provision of standardized test meals or standardized foods/meals administered at various time points throughout the study in congruence with collecting metabolomics data may assist with evaluating individual responses. Overall provision of partial or whole diet can also reduce variability in food preparation or cooking practices.

#### DID29: Acknowledgment of the generalizability of the population being studied

Recommendation: Described in 1 sentence or very briefly.

Example:

…the inpatient environment of the metabolic ward makes it difficult to generalize our results to free-living conditions. However, current dietary assessment methods are insufficient to accurately or precisely measure energy intake outside the laboratory [87,88], and adherence to study diets cannot be guaranteed in free-living subjects. [41], p. 75.

Explanation: Although generalizability may be covered by other reporting guidelines, it should be reported in a feeding study examining the dietary metabolome to assist with the emergence of the field of metabolomics, where cultivation of population-specific findings is key for global advancement of the field. Generalizability could be reported in the methods or discussion/conclusion sections of research papers.

An example of a completed DID-METAB Checklist has been included for a published protocol article, published by the PPN team (Supplemental Table 5). In this example, DID18 and DID20 were completed as “N/A—protocol paper” as it is more applicable to report these DIDs in an outcomes paper. The DID-METAB Checklist indicated that DID8 and DID29 were inadequately reported in this article, and thus the personnel (and their relevant qualifications) responsible for designing the dietary interventions as well as the prospective generalizability of the population being studied should have been reported. Moreover, additional information and/or examples in the supplementary file for DID3, DID6, DID7, DID10, DID11, DID19, DID21, and DID24 could have also been provided.

## Conclusions

A COS was identified to improve reporting of dietary interventions administered within controlled human feeding studies designed to measure the metabolome and will likely have utility for human feeding studies investigating other outcomes, for example, microbiome studies. The DID-METAB Checklist has been developed to assist authors with reporting on these interventions in a replicable manner. The tool also assists reviewers and editors to evaluate adequacy of methodologic descriptions, researchers when interpreting and utilizing the information, while helping advance the implementation of metabolomics in nutrition research by enhancing standardization of dietary intervention methodology reporting, ultimately facilitating evidence synthesis. This framework will enhance the utilization of metabolomics in nutrition studies through the following: *1*) development of minimum core details related to the methodology of dietary feeding interventions; *2*) delineation of the minimum reporting procedures required in the manuscript or protocol; *3*) facilitating consistency of methods and data reported across feeding interventions allowing increased reproducibility; *4*) enhancing data quality, interoperability, and reusability; *5*) reducing unnecessary methodologic and/or reporting heterogeneity across studies, and *6*) improving evidence synthesis and/or meta-analyses in metabolomics.

Similar to other reporting guidelines, we defined a minimum core set of items identified using a Delphi technique and translated these into recommendations, a checklist, and an explanatory document, enhanced using real-world examples. Some limitations that should be acknowledged is that although the expert sample size in this Delphi may be viewed as comparably smaller to some used in the development of other reporting guidelines [19,89,90], there is no published agreement on the optimal size of an expert panel, and ours aligns with those commonly undertaken in the field of health sciences [23]. Moreover, the scope of the project was focused on improving the reporting and reproducibility of methodologies relating to feeding interventions and the nutritional metabolome only, thus the DID-METAB Checklist, although it could be used as a stand-alone tool in relevant studies, does not address reporting guidelines or recommendations for other sections of research articles (e.g., introduction, results, and discussion) nor methodologic aspects specific to biospecimen handling and preparation and/or metabolomics analysis techniques. A notable strength of this Delphi is that it involved an international group of experts, with a collective >200 y of research expertise across relevant fields, and a core research team with extensive expertise in dietary assessment methodologies, clinical and experimental research design, and implementation of Delphi techniques who designed the project and reconvened for consensus after every Delphi round. Other strengths include the identification of a COS according to minimum standards [22], development of the checklist guided by recommendations for the development of reporting guidelines [18], high response rate (88%), retention of all 22 international experts in the final 3 rounds, broad caliber of expertise, and unanimous consensus on the final version of the checklist.

Given the DID-METAB Checklist concerns only the methodologic aspects of the dietary intervention component of human feeding studies, we strongly suggest that authors submitting RCTs also use this in parallel with the CONSORT 2010 Statement checklist [80], integrating the DID-METAB Checklist at item 5 of the CONSORT checklist. Likewise, for authors submitting reports of clinical trial protocols, we suggest authors integrate the DID-METAB Checklist at item 11 of the SPIRIT 2013 checklist [91]. A similar approach has been recommended with the application of other reporting guidelines such as the TIDieR (Template for Intervention Description and Replication) checklist, which was devised to improve the reporting of interventions to enhance replicability. The authors recommend application of the TIDieR checklist with both the CONSORT and SPIRIT checklists, where relevant [89]. Notably, the Federation of European Nutrition Societies has recently recommended that a nutrition extension to the CONSORT statement (CONSORT-Nut) be devised to address the gap in rigorous and standardized reporting of human nutrition intervention trials [17,92]. The international working group identified 28 new nutrition-specific recommendations for reporting items in the introduction (3), methods (12), results (5) and discussion (8) sections of a nutrition research article [17]. Although 12 new nutrition-specific recommendations for the methodology of nutrition trials have been identified as part of the proposed CONSORT-Nut, these items are generalized and not specific to nutrition trials where the dietary metabolome is examined. Therefore, it is vital that reporting of such studies is done in a rigorous and replicable manner as instructed in the DID-METAB Checklist, in order to advance the rapidly growing field of nutritional metabolomics and its application to precision and personalized nutrition.

Journals are encouraged to endorse the use of the DID-METAB Checklist as a key strategy for enhancing both the implementation of the checklist and increased rigor in study design and reporting of human feeding studies measuring the dietary metabolome. This can be achieved by incorporating this recommendation into author guidelines, listing a URL link to the checklist or EQUATOR network, and/or publishing an editorial announcement about the journal’s endorsement of the reporting tool and to what articles it may be relevant. To encourage dissemination and use of a single standard for reporting, the DID-METAB Checklist has been simultaneously published in the *European Journal of Clinical Investigation*.

Unlike existing checklists, the DID-METAB Checklist provides recommendations around the level of detail expected for various items, which may also assist with the prioritization of the location of reporting information such as manuscript methodology, summary table/figure, or a supplementary file. A recurring theme of commentary from experts in the current Delphi acknowledged that these reporting recommendations vary based on study design, complexity of dietary interventions, journal requirements or restrictions, and whether the article is an outcomes paper or protocol/methodology paper. Hence, why this consideration has been added to the checklist to tailor the usability for authors.

Evaluation of the impact of implementation of the DID-METAB Checklist on reporting future feeding studies examining the nutritional metabolome will be important moving forward, as shown for other similar reporting tools [93]. As the field of nutritional metabolomics continues to evolve and its application in precision and personalized nutrition grows, evaluation and extension of these recommendations are warranted, and the checklist should be reappraised periodically.

## Author contributions

The authors’ responsibilities were as follows – JJAF, EDC, JS, MG-M, TJ, CEC: conceptualized and designed the research; JJAF, TJ: collected and assembled the data; JJAF, analyzed and collated the data, presenting it to EDC, JS, and MG-M after each stage of the Delphi process to incorporate expert feedback; TJ: provided administrative and technical support for the Delphi; JJAF: wrote the paper; EDC, JS, MG-M, TJ, and CEC: involved in critical revision of the paper; JJAF: had primary responsibility for final content; and all authors: have read and approved the final manuscript. All members of the DID-METAB Delphi Working Group were participants of the entire 2-stage Delphi process and thus contributed to data collection, development of the checklist, and reviewed this paper as co-authors.

## Funding

This work and CEC were supported by a National Health and Medical Research Council (NHMRC) Leadership Investigator grant (APP2009340), who had no input in any aspect of the project. All DID-METAB Delphi Working Group members were volunteers.

## Conflict of interest

CEC reports financial support was provided by National Health and Medical Research Council (NHMRC, APP2009340). EH is supported by a Laureate Fellowship from the Australian Research Council and is a director of Melico, outside the scope of the submitted work with no financial contribution. FZM is supported by a Senior Medical Research Fellowship from the Sylvia and Charles Viertel Charitable Foundation, a National Heart Foundation Future Leader Fellowship (105663), and NHMRC Emerging Leader Fellowship (GNT2017382). MS is supported by a National Heart Foundation Postdoctoral Fellowship (106698). JJAF holds a separate part-time employment at Sanitarium Health Food Company who had no input into the study and are not financially supporting or sponsoring any part of this study. MHT is supported by the BBSRC Core Capability Grant BB/CCG2260/1 and its constituent project BBS/E/QU/23NB0006 (Food and Nutrition National Bioscience Research Infrastructure). MAM is an Editorial Board Member for *Advances in Nutrition* and played no role in the Journal’s evaluation of the manuscript. All other authors report no conflicts of interest.
