# Supplementary material for: Diet Item Details: Reporting Checklist for Feeding Studies Measuring the Dietary Metabolome (DID-METAB Checklist)—Explanation and Elaboration Report on the Development of the Checklist by the DID-METAB Delphi Working Group
Source: Adv Nutr. 2025 Apr 14;16(5):100420. doi: 10.1016/j.advnut.2025.100420 (PMC12076792; doi:10.1016/j.advnut.2025.100420)
Supplement: Multimedia component 1 [file mmc1.docx]

**Supplementary Methods –** Delphi survey and consensus generation.

*Stage 1*

Round 1 of the first stage asked participants to select ‘include’ or ‘exclude’ to identify core DIDs. For DIDs voted as ‘include’, participants were prompted to select a binary option indicating whether they ‘agree’ or ‘disagree’ with the wording or phrasing. If they selected ‘disagree’, they were given the opportunity to provide a reason and/or propose amendments to phrasing in a free text field. The opportunity to suggest additional DIDs for each domain was also provided. All DIDs that reached consensus including any new DIDs proposed by the expert panel were reviewed by the PPN team and any deemed as a new or novel DID, were carried forward to round 2.

In round 2, participants were asked to vote on DIDs as well as the wording or phrasing for the last time using the same binary question format in round 1. Participants were provided with the opportunity to comment only at the end of each set of domain questions. All DIDs voted as ‘include’ by consensus were carried forward to stage 2.

*Stage 2*

Stage 2 asked participants in round 1 to select from a series of options both the location of DID reporting in a research manuscript and the level of detail of reporting. For each core DID, participants were asked to select if it should be reported in: ‘description provided within methods’, ‘additional table’, ‘additional figure’, or ‘supplementary file’. Participants were able to select as many that they believed applied for that DID with the opportunity to provide *ad libitum* commentary such as the title of a table, figure caption etc. To assist with interpretation, examples of the level of detail of reporting accompanied each option (**Supplementary Methods Table 1**). An optional ad libitum field to provide references or examples to support their suggestions was also provided. Participants were provided with the opportunity to comment at the end of each domain. All DID reporting recommendations were initially analysed and commentary thematically summarised by the lead investigator (JJAF). Results were then presented to at least two or more PPN Team members (EC, JS, MGM) for discussion. A consensus was reached on the criterion to establish a hierarchy of reporting recommendations for each DID. Core DIDs and their reporting recommendations were carried forward to round 2.

**Supplementary Methods Table 1 –** Examples of the level of detail for reporting provided to experts to help guide their commentary in stage 1 round 1.

| **Location of reporting in research paper** | **Example of the level of detail of reporting^1^** |
| --- | --- |
| **Description provided within methods** | Describe in only a couple of sentences; describe in detail up to ~ 250 words; describe in detail in a separate paragraph under its own sub-heading titled ‘XX’; provide calculations for formulas where relevant |
| **Additional table** | Report in a table titled ‘XX’; report in a table separately for each intervention arm and/or total population; include references where relevant in table footnotes. |
| **Additional figure** | Describe in a figure titled ‘XX’; describe in separate figures for each intervention arm and/or total population; include references where relevant in figure footnotes. |
| **Supplementary file** | Describe in a table/figure; describe in detail in a series of table(s)/figure(s); provide a copy of [insert relevant documentation name e.g., participant resource, tool used, calculations, formulas]. |
| **Where appropriate, include references (optional)** | Include links to exemplar papers or sources that can serve as guides to set expectations for the necessary level of detail, or the minimum standard required. This would offer a valuable reference point. |

^1^ These were made available as a reference for experts every time they were asked to vote on the location of reporting and provide comment on the level of detail of reporting. Experts were notified that if they left the comment field blank, the first example provided in the list was adopted as their default response.

In round 2, participants were asked to ‘agree’ or ‘disagree’ with each DID reporting recommendation (which included presenting the DID, the location of the manuscript and supplementary material where each DID should be reported, and level of detail of reporting). Participants were able to provide ad libitum commentary at the end of each domain. All DID recommendations that met consensus were carried forward to round 3 where they were formatted into a table (i.e., reporting checklist now called DID-METAB Checklist).

In round 3, participants were asked in binary format to ‘accept’ or ‘not accept’ the checklist. In the case of ‘not accept’, participants were given the opportunity to include comments explaining their decision. Participants were also asked in binary format, questions about whether they would use the checklist in their research, if they support the recommendation for the checklist to be used alongside existing tools where relevant, whether relevant journals should recommend the use of the checklist for relevant studies, and if they intend to use the checklist in future studies they may be involved in and why. Open-ended questions were asked to gather general commentary around suggestions for implementation and dissemination of the checklist. Experts were asked to accept or decline invitation to be a co-author under the DID-METAB Delphi Working Group on associated manuscripts relating to the reporting guideline development.

*Generating a consensus*

A cut-off agreement rate of 70% was employed to make a decision about each DID and reporting recommendations for binary questions. This rate is similar to what has been employed in other studies (1). There is no uniform definition of consensus; however, a threshold of 60% or higher appears to be used in most Delphi methodologies in health sciences (2). On occasions where a consensus rating of < 70%, but ≥ 50% was returned for the inclusion of a DID during stage 1, if the associated consensus rating for the phrasing was ≥ 70%, the DID was included for re-phrasing via incorporation of expert feedback (where available) as well as via discussion amongst the PPN team. This is because since the phrasing achieved consensus, the PPN team believed minor amendments to re-phrasing to provide clarity may assist with interpretation of the DID, and thus, subsequent consensus for inclusion after testing in the next round (**Supplementary Methods Figure 1**). Since an approval voting style was used in stage 2 round 1, the PPN team devised and implemented criterion to inform the allocation of a hierarchy of recommendations for reporting level of each DID (**Supplementary Methods Table 2**). Criterion considered both the percentage of votes for the location reporting, and number of relevant expert commentaries.

Issues raised by participants, such as suggestions for new DIDs, changes to wording, or recommendations for reporting, were reviewed by the PPN team after each round in a roundtable meeting. Feedback was discussed, and the PPN team devised appropriate changes, such as rewording, combining, or splitting core DID phrasing and reporting recommendations, which were then incorporated into subsequent surveys for further expert review. PPN roundtable meetings and revisions were co-ordinated by the lead investigator (JJAF).

**Supplementary Methods Figure 1 –** Decision tree for criteria applied during stage 1 round 1 of the Delphi.

Stage 1 round 1

No

Yes

No

Yes

DID entered stage 1 round 2 with no essential changes required

DID entered stage 1 round 2 with changes required

DID discarded from the Delphi

No

Yes

Did the DID receive ≥70% consensus for phrasing?

Did the DID receive ≥50% consensus for inclusion as core AND ≥70% consensus for phrasing?

Did the DID receive ≥70% consensus for inclusion as core?

**Supplementary Methods Table 2 –** Criterion used to establish a consensus on DID reporting sets in stage 2 round 1.

| **Result from Stage 1 Round 1** | **Action** | **Reporting set terminology to employ** |
| --- | --- | --- |
| 1 vote, no comment or irrelevant commend made^1^ | Discard from reporting set | N/A |
| 1 vote, with relevant comment supporting its inclusion | Include in reporting set | “Optional” |
| < 10% vote and ≥ 50% comments supporting its inclusion | Include in reporting set | “Optional” |
| 10 – 24% vote and ≥ 50% comments supporting its inclusion | Include in reporting set | “Consider” |
| ≥ 25% vote and ≥ 50% comments supporting its inclusion | Include in reporting set | “Recommendation” |
| ^1^ ‘Vote’ applies to the location of reporting, and ‘comment’ refers to commentary provided in relation to the level of detail. | | |

**Supplementary Figure 1 –** Flow of experts in the Delphi consultation rounds.

67 experts invited to participate in the study

**Stage 2 Round 3**

N=22 experts completed the survey and thus entire Delphi

**Stage 2 Round 2**

N=22 experts completed the survey and invited to participate in Stage 2 Round 3

**Stage 2 Round 1**

N=22 experts completed the survey and invited to participate in Stage 2 Round 2

3 experts were non-respondents

4 experts were non-respondents

**Stage 1 Round 2**

N=25 experts completed the survey and invited to participate in Stage 2 Round 1

**Stage 1 Round 1**

N=25 experts completed the survey and invited to participate in Stage 1 Round 2

Ineligible (n=1)

Insufficient expertise (<1 year expertise in relevant research area)

N=29 invited to take part Stage 1 Round 1

N=30 completed online eligibility screening survey and provided consent

**Supplementary Table 1 –** Characteristics of experts who completed the Delphi (n=22).

|  | N (%) |
| --- | --- |
| Age bracket  18-24 years  25-34 years  35-44 years  45-54 years  55-64 years  Above 64 years | 0 (0%)  1 (5%)  9 (40%)  5 (23%)  6 (27%)  1 (5%) |
| Sex  Female  Male | 18 (82%)  4 (18%) |
| Current geographic location  Australia  United States  United Kingdom  New Zealand  Israel  Italy  Denmark  Sweden | 11 (50%)  3 (14%)  3 (14%)  1 (5%)  1 (5%)  1 (5%)  1 (5%)  1 (5%) |
| Expertise area^1^  Clinical and experimental design of dietary interventions  Clinical and experimental design of human feeding studies (partial/whole diet provision)  Dietary metabolomics  Diet-related biospecimen analyses and interpretation  Other^2^ | 21 (95%)  20 (91%)  13 (59%)  15 (68%)  9 (41%) |

^1^ Majority of experts declared expertise in more than one area, therefore, results are summarised accordingly and percentages per category will not equate to 100.

^2^ Other areas of expertise that were listed were: non-communicable disease (n=1); hair metabolomics (n=1); food metabolomics (n=1); dietary assessment (n=2); body composition assessment (n=2); physical activity assessment (n=1); effects of eating environment and meal timing on dietary intake (n=1); nutritional epidemiology (n=1); development and implementation of biomarkers of food intake applications of metabolomics in doping studies (n=1); deep phenotyping of individuals with metabolic disease (n=1); muscle and plasma lipidomics (n=1), algorithm-assisted dietary plans based on big data (n=1); energy expenditure, body composition, appetite regulation, stable isotopes, postprandial metabolism (n=1); analytical chemistry analysing dietary interventions and biological samples for outcome (n=1); clinical biomarkers, lipidology, and preventive cardiology (n=1); and investigating the microbiome including datasets such as metagenomics and metabolomics in response to dietary interventions (n=1).

**Supplementary** **Table 2 –** DIDs to be considered core including their phrasing as identified by reaching consensus at the end of stage 1 round 2.

|  | **Round 1** | |  | **Round 2** | |
| --- | --- | --- | --- | --- | --- |
| **DID** | **Include^1^** | **Phrasing^2^** | **DID** | **Include** | **Phrasing** |
| **1** | 100.00% | 68.00% | **1** | 96.00% | 96.00% |
| **2** | 80.00% | 95.00% | **2** | 84.00% | 84.00% |
| **3** | 96.00% | 75.00% | **3** | 92.00% | 84.00% |
| **4** | 96.00% | 79.10% | **4** | 96.00% | 88.00% |
| **5** | 52.00% | 84.62% | **5** | 72.00% | 92.00% |
| **6** | 92.00% | 86.96% | **6** | 96.00% | 84.00% |
| **7** | 100.00% | 76.00% | **7** | 100.00% | 80.00% |
| **8** | 68.00% | 70.59% | **8** | 72.00% | 76.00% |
| **9** | 100.00% | 84.00% | **9** | 96.00% | 84.00% |
| **10** | 96.00% | 83.33% | **10** | 100.00% | 96.00% |
| **11** | 76.00% | 84.21% | **11** | 88.00% | 84.00% |
| **12** | 92.00% | 73.91% | **12^^^** | 88.00% | 88.00% |
| **13** | 68.00% | 94.12% |  |  |  |
| **14** | 84.00% | 76.19% | **13** | 84.00% | 88.00% |
| **15** | 100.00% | 88.00% | **14** | 96.00% | 92.00% |
| **16** | 100.00% | 60.00% | **15** | 96.00% | 96.00% |
| **17** | 88.00% | 72.73% | **16** | 96.00% | 100.00% |
| **18** | 84.00% | 71.43% | **17** | 84.00% | 92.00% |
| **19** | 92.00% | 86.96% | **18** | 96.00% | 92.00% |
| **20** | 100.00% | 96.00% | **19** | 92.00% | 96.00% |
| **21** | 96.00% | 87.50% | **20** | 92.00% | 92.00% |
| **22** | 100.00% | 92.00% | **21** | 96.00% | 100.00% |
| **23** | 96.00% | 95.83% | **22** | 100.00% | 100.00% |
|  |  |  | **23^†^** | 92.00% | 96.00% |
| **24** | 80.00% | 85.00% | **24** | 84.00% | 88.00% |
| **25** | 92.00% | 91.30% | **25** | 92.00% | 92.00% |
| **26** | 100.00% | 96.00% | **26** | 100.00% | 96.00% |
| **27** | 72.00% | 77.78% | **27** | 92.00% | 92.00% |
| **28** | 92.00% | 78.26% | **28** | 92.00% | 92.00% |
|  |  |  | **29^†^** | 88.00% | 100.00% |
| Experts asked to vote whether to ‘include’ or ‘exclude’ the DID as well as whether they ‘agree’ or ‘disagree’ with the phrasing of the DID. DIDs are listed in the table accordingly to enable ease of comparison of consensus ratings, accounting for the merging of two DIDs, and the addition of 2 new DIDs.  ^^^ DID was rephrased to merge DID12 & DID13 from round 1.  ^†^ New DIDs identified from Stage 1 round 1. | | | | | |

**Supplementary** **Table 3 – Example of recommended information to be included in a supplementary file for DID7**
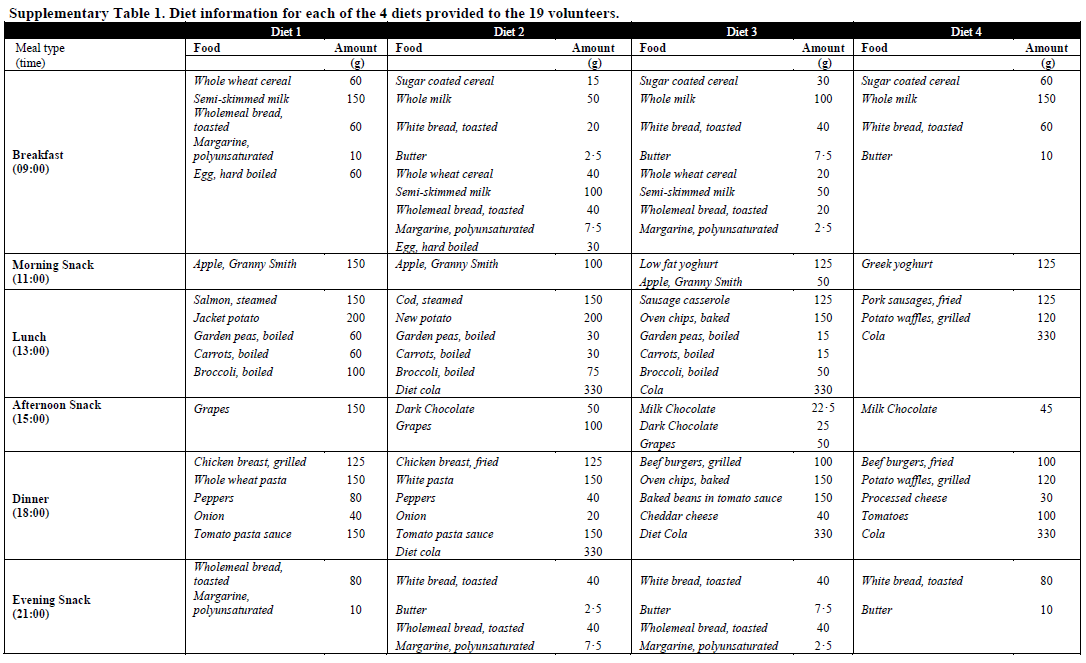
**.**

**Source:** Garcia-Perez I, Posma JM, Gibson R, Chambers ES, Hansen TH, Vestergaard H, Hansen T, Beckmann M, Pedersen O, Elliott P, Stamler J, Nicholson JK, Draper J, Mathers JC, Holmes E, Frost G. Objective assessment of dietary patterns by use of metabolic phenotyping: a randomised, controlled, crossover trial. Lancet Diabetes Endocrinol. 2017 Mar;5(3):184-195. doi: 10.1016/S2213-8587(16)30419-3. Epub 2017 Jan 13. PMID: 28089709; PMCID: PMC5357736 (3).

**Supplementary** **Table 4 – Example of recommended information to be included in a supplementary file for DID 10.**

Supplementary Table 1. Foods provided weekly* to volunteers in the two groups by study center.

| **Center** | **Intervention group** | **Control group** |
| --- | --- | --- |
| Aarhus (Denmark) | - whole-grain rye or wheat bread  - whole-grain rye crispbread*  - oat flakes, rye flakes or muesli*  - frozen berries (mixed)*  - whole berries (cranberries, blackcurrants or raspberries)  - dried berry powder (blueberry or blackcurrant)*  - fish (tuna, herring, salmon or mackerel)  - nuts (hazelnuts, almonds, walnuts, pine nuts)*  - yoghurt (3.5% fat) based on *Lactobacillus acidophilus*.  - low-fat cream or creamer (both 7% fat)*  - margarine (38% fat) with high PUFA content (15%)* | - refined wheat bread  - rice crackers and wheat crackers with sesame seeds or cinnamon*  - yellow or chocolate cake  - corn flakes*  - semolina, refined pasta and polished rice*  - jams and marmalades with mixed berries, strawberries or orange*  - honey*  - butter or butter-based spread  - low-fat creamer (15-20% fat)* |
| Kuopio (Finland) | - rye bread (whole grain rye flour, crushed and whole grains, rye bran, yeast, sea salt, water)  - rye bread of peeled rye; peeled rye flour, wheat flour, yeast, salt, water  - whole grain pasta; whole grain durum wheat, durum wheat, water*  - oat flakes (wholegrain)*  - frozen bilberries or strawberries*  - frozen bilberry puree (mashed bilberries and sugar)*  - bilberry, blackcurrant, or strawberry powder (pure dried berries, all parts included)*  -vegetable oil based spread, liquid margarine and rapeseed oil* | - toast bread (wheat flour, crushed whole grain wheat, yeast, syrup, rapeseed oil, salt)  - wheat bread (wheat flour, water, peeled rye flour, yeast, potato flakes, salt, wheat gluten, rapeseed oil, syrup, rye malt)  - wheat bread (wheat flour, yeast, salt, water, wheat protein, rapeseed oil, sorbate)  - carrot rolls (wheat flour, carrot, whole grain wheat flour, water, yeast, syrup, salt, oat fiber, rapeseed oil, wheat gluten)  - wheat rolls; wheat flour, water, vegetable fat, salt, mono- and diglycerides of fatty acids)  - wheat oat bread (wheat flour, crushed oats, oatmeal, oat bran, wheat germ, sugar, enzyme, ascorbate, water, salt, yeast)  - wheat bread with rye malt (wheat flour, rye malt, wheat germ, sugar, enzyme, ascorbate, water, salt, yeast)  - bagel (wheat flour, water, sugar, vegetable oil, yeast, salt, barley malt extract, soya lecithin, calcium acetate and propionate)  - rice (white)*  -butter-vegetable oil spread* |
| Lund (Sweden) | -margarine based on sunflower, linseed and rapeseed oils  -whole-grain rye and wheat bread  -oat beverage, whole-grain pasta  -rye and oat crisp bread, wholegrain rusks*  -frozen strawberries and berry powders (bilberrry, strawberry, black currant)*  -walnuts, hazelnuts, almonds, sunflower seeds, linseeds  -green peas and yellow peas, kidney beans*  -muesli, porridge oats, oat and barley seeds, red lentils, bilberry jam* | -butter, butter oil  - margarine*  -low-fiber-wheat bread, wheat rusks  -sausages, low-fiber pasta  -rice, corn flakes, semolina*  -whipping cream, cinnamon pastry, sponge cake  -honey, orange jam, apricot jam* |
| Oulu (Finland) | - rye bread (whole grain rye flour, crushed and whole grains, rye bran, yeast, sea salt, water)*  - rye bread of peeled rye; peeled rye flour, wheat flour, yeast, salt, water  - whole grain pasta; whole grain durum wheat, durum wheat, water*  - oat flakes (wholegrain)*  - frozen bilberries or strawberries*  - frozen bilberry puree (mashed bilberries and sugar)*  - bilberry, blackcurrant, or strawberry powder (pure dried berries, all parts included)*  - vegetable oil based spread, liquid margarine and rapeseed oil* | - toast bread (wheat flour, crushed whole grain wheat, yeast, syrup, rapeseed oil, salt)  - wheat bread (wheat flour, water, peeled rye flour, yeast, potato flakes, salt, wheat gluten, rapeseed oil, syrup, rye malt)  - wheat bread (wheat flour, yeast, salt, water, wheat protein, rapeseed oil, sorbate)  - carrot rolls (wheat flour, carrot, whole grain wheat flour, water, yeast, syrup, salt, oat fiber, rapeseed oil, wheat gluten)  - wheat rolls; wheat flour, water, vegetable fat, salt, mono- and diglycerides of fatty acids)  - wheat oat bread (wheat flour, crushed oats, oatmeal, oat bran, wheat germ, sugar, enzyme, ascorbate, water, salt, yeast)  - wheat bread with rye malt (wheat flour, rye malt, wheat germ, sugar, enzyme, ascorbate, water, salt, yeast)  - bagel (wheat flour, water, sugar, vegetable oil, yeast, salt, barley malt extract, soya lecithin, calcium acetate and propionate)  - rice (white)*  - butter-vegetable oil spread* |
| Reykjavik (Iceland) | - Bilberry powder (pure dried bilberry, all parts of the berry included) (same product as used in Finland)*  - Strawberry powder (pure dried strawberry, all parts of the berry included) (same product as used in Finland)*  - Barley (from The Mother Earth Farm at Vallanes)*  -Spread from Unilever (same product as used in Sweden and Denmark)* | - None |
| Uppsala (Sweden) | - Bilberry, blackcurrant and strawberry powder (pure dried berries, all parts included)*  - Rye crispbread (100% rye wholemeal)*  - Wheat and rye bread (containing wheat, rye flakes, rye wholemeal sourdough, rapeseed oil, linseeds, rye fibre). 21% of grain is rye.  - Rye bread (containing wholemeal rye, rye wholemeal sourdough, wheat, rye flakes, rapeseed oil). 66% of grain is rye. 38% wholemeal (73% of dry weight)  - Rye bread (containing wholemeal rye, rye wholemeal sourdough, wheat wholemeal, rye fibre). 83% of grain is rye. 53% wholemeal (100% of dry weight)  - Grain flake mix (rye, wheat, oat, barley)*  - Pearl barley*  - Muesli with fruits and hazelnuts (containing oat flakes, wheat flakes, raisins, apricots, rye flakes, apples, hazelnuts)*  - Oat bran*  - Wheat bran*  - Wheat kernels (100% wholegrain)*  - Oat kernels (100% wholegrain)*  - Barley kernels (100% wholegrain)*  - Scones baked with rapeseed oil  - Pasta, 55% wholemeal*  - Pasta with extra wheat fibre*  - Margarine (per 100g: SFA 12 g, MUFA 17 g, PUFA 31 g)*  - Rapeseed oil*  - Hazelnuts*  - Sunflower seeds* | - White wheat crisp bread*  - White wheat bread (containing wheat and rye, syrup, sugar, rapeseed oil, rye fibre)  - White wheat soft thin bread (containing wheat and wheat wholemeal, rapeseed oil, sugar)  - French hot dog bread (white wheat)  - Muesli (based on oats; 20 g sugar per 100 g of product)  - Semolina*  - Muesli with tropic fruits (oat flakes, raisins, wheat flakes, rye flakes, cornflakes, ananas, barley flakes, banana chips, sugar, honey)*  - Wheat crusts*  - Scones baked with butter  - White pasta*  - Butter |

Grain, spread/oil and berry products were supplied to fulfill the calculated need for each volunteer during the week while fresh fish were bought by the volunteers at most centers and reported. * Foods (staples or frozen) that were sometimes provided with larger intervals (up to one month).

**Source:** Gürdeniz G, Uusitupa M, Hermansen K, Savolainen MJ, Schwab U, Kolehmainen M, et al. Analysis of the SYSDIET Healthy Nordic Diet randomized trial based on metabolic profiling reveal beneficial effects on glucose metabolism and blood lipids. Clin Nutr. 2022 2022/02/01/;41(2):441-51 (4).

**Supplementary Table 5 – Diet Item Details: Reporting checklist for feeding studies measuring the human dietary metabolome (DID-METAB Checklist) -** completed for Ferguson JJA, Clarke E, Stanford J, Burrows T, Wood L, Collins C. Dietary metabolome profiles of a Healthy Australian Diet and a Typical Australian Diet: protocol for a randomised cross-over feeding study in Australian adults. BMJ Open. 2023 Jul 31;13(7):e073658 (5).

| **Details to include when describing the methodology of feeding studies and the appropriate sections for reporting this information.** | | | |
| --- | --- | --- | --- |
| *The DID-METAB Checklist is for reporting dietary details used in intervention and control groups in human feeding studies related to the dietary metabolome. The aim is to ensure adequate reporting of dietary methodology and to facilitate replication. Other study components are covered by existing reporting statements and checklists. Further information is included in the DID-METAB guide paper and should be used alongside the DID-METAB Checklist.* | | | |
| Grouped under five Domains, are 29 Diet Item Details (DIDs) with a hierarchy of reporting recommendations. Those labelled as ‘consider’ or ‘optional’ are additional suggested recommendations that may guide the methodology choices of study design. Examples of content to report for each DID are also provided in the table.  For each DID reporting recommendation, please specify where it is documented by indicating the manuscript page number, supplementary materials or other resources (e.g., protocol paper or pre-print) in the last (where reported) column. If a DID is not applicable to the intervention or study design, please use ‘N/A’.  It is strongly recommended that this checklist is used in conjunction with the CONSORT 2010 Statement1, as an extension of Item 5 when a randomised clinical feeding trial is being reported, or in conjunction with the SPIRIT 2013 Statement2 as an extension of Item 11 for clinical feeding trial protocols. DID-METAB Checklist can also be used in conjunction with checklists relevant to other study designs (see www.equator-network.org). While the DID-METAB Checklist is intended for the methods section of a paper (unless explicitly stated as ‘supplementary file’), in some cases specific items may be more relevant to be reported in other sections, e.g. results or discussion. | | | |
| **DID no.** | **Diet Item Detail (DID)** | **Recommendations for reporting item** | **Where reported^†^**  *Page no. or Supplementary no.* |
| **DOMAIN 1 – DIETARY INTERVENTION – MODELLING** | | | |
| **1** | **Methods and/or tools used to design the nutritional/dietary characteristics of the dietary intervention(s) and control diet(s) employed.**   - *Detailed methods reported to replicate a published position or well-established therapeutic diet or dietary trend such as DASH, Mediterranean Diet e.g., <X mg sodium, X% sat fat (X serves of fruits and vegetables) etc, including references.* - *Software used including version number e.g., ProNutra ver 1.0* | Detailed description (up to ~250 words) | 3-4 |
|  |  | Detailed description for novel or non-standard method and/or tools and/or if journal is non nutrition/dietetic in a supplementary file.  Provide an example of method/tools in a supplementary file. | N/A |
|  |  | **Optional:** describe in a table | N/A |
| **2** | **References to population-based dietary guidelines, survey data and/or published therapeutic diets (where possible) that inform the design of dietary interventions.**   - *National or International population-based dietary guidelines* - *National survey data* | Brief description (couple of sentences) | 3-4 |
|  |  | **Consider:** detailed description in a supplementary table. | 4 – Table 1 |
| **3** | **Method(s) used for personalising and/or modifying the dietary intervention(s) and control diet(s). This may include implementing dietary substitutions to accommodate specific diet or nutritional needs; individual preferences; anthropometric, biochemical or clinical profile; and/or product availability/seasonality.**   - *Energy matching dietary intervention by upscaling or downscaling food items according to participant’s basal energy intake OR calculated energy requirements* - *Food/meal substitutes due to food allergies, intolerances, aversions, or specific nutritional requirements* | Brief description (couple of sentences) | 4 |
|  |  | Detailed description in a supplementary table(s), figure(s) and/or provide examples. | Not reported |
|  |  | **Optional:** describe in a table | Not reported |
| **4** | **a) Food composition database and/or reference material used to analyse the nutritional content of the dietary intervention(s) and control diet(s), including references.**   - *Australian Food Composition Database (e.g., AUSNUT 2013 formerly NUTTAB)* - *Software programs used including reference to version number e.g., FoodWorks, ProNutra* | Brief description (couple of sentences) | 6 |
|  |  | **Consider**: detailed description in supplementary table. | Not reported |
| **5** | **b) Details of the applicability of the food composition database and/or reference material to the population being studied.**  *Explanation of how the food composition database is representative of the population being studied, including references. Or, if the database used is not representative of the population, explain why it was used and/or why it was considered the best available or an appropriate substitute.* | Brief description (couple of sentences) | 3 |
| **6** | **Method(s) used to standardise dietary intake within groups.**   - *Food library reference with pre-determined food/meal substitutes for each dietary intervention.* - *Full (or at least partial) provision of foods, meals and/or raw ingredients.* - *Where food is supplied, the following may be relevant: grocery order placed by study investigators, meals made in test/commercial kitchen, participants required to consume X number of meals at research facility under supervision, participants to collect foods from research site X times per week, minimal food preparation or cooking required.* - *Identical meal plans provided to participants* - *Support resources e.g., foods/meals to choose when eating out, takeaway for each dietary intervention* - *Description of food form e.g., mashed, pieces, powder* | Detailed description (up to ~250 words) | 4-5 |
|  |  | Detailed description in a supplementary table(s) | Not reported |
| **7** | **Qualitative and quantitative characteristics of all dietary intervention(s) described in a reproducible manner.**   - *Portion sizes, required serves per food group, food choices/characteristics e.g., beta-carotene-rich fruits and vegetables, wholegrain vs refined grain products etc* - *Nutrient targets* - *Example meal plan, or rotating menu* - *Timing of food intake, food/meal patterns* | Detailed description in a separate paragraph under its own subheading | 3-4 |
|  |  | Provide example meal plan or rotating menu in a supplementary file | Not reported |
|  |  | **Consider:** detailed description in table for each diet group | 4 – Table 1 |
| **8** | **Personnel responsible for designing and developing the dietary intervention(s) and control diet(s); including who developed menu/meal plans; provided dietary education; and any documents/resources provided to the participants clearly identified along with their relevant qualifications.**   - *Research Dietitian, Registered Nutritionist/Dietitian, Accredited Practising Dietitian, research team member in liaison with any of the aforementioned.* - *Or list relevant qualifications, certifications, training undertaken and/or experience for personnel involved.* | Brief description (couple of sentences) | Not reported |
|  |  | Detailed description and/or provide documentation of participant resources in supplementary file | Not reported |
| **DOMAIN 2 – DIETARY INTERVENTION - IMPLEMENTATION** | | | |
| **9** | **The proportion of food and/or beverages provided for each dietary intervention.**   - *All or full provision of diet should be stated or inferred* - *Partial or expressed as a % or proportion of total food intake e.g., 80% or 90% of all foods and beverages needed for individual consumption were provided to participants* - *Provision of any key food items relevant to the dietary intervention(s) e.g., provision of olive oil for a Mediterranean diet* - *If relevant, provide specific weight of food(s) provided e.g., 100g berries* - *Description of any food allowances e.g., condiments, spices, seasonings, water, noncaloric beverages etc* | Brief description (couple of sentences) | 1, 4 |
|  |  | **Consider**: detailed description in a supplementary table(s) for each diet group and/or examples of participant handouts/resources provided | Not reported |
| **10** | **Nature of the food and/or beverages provided (e.g., recipe of test food/meal, raw ingredients, cooking instructions, pre-prepared meals, combination etc), storage conditions, and how this was provided to participants (e.g., delivered to their home, fed onsite, collected from supermarket).**   - *Raw ingredients provided which participants used to assemble / cook own meals; only pre-prepared/cooked meals provided; combination or raw ingredients and pre-prepared meals.* - *Participants collected grocery order from supermarket or research facility, or study food was delivered to participants’ house, or participants were provided with a gift card to purchase groceries etc.* - *Foods prepared by a research test kitchen, third-party quality-controlled kitchen, or commercial kitchen to ensure standardisation* | Brief description (couple of sentences) | 4 |
|  |  | Detailed description in a supplementary table(s) and/or figure(s) where applicable and/or examples of participant handouts/resources provided | Not reported |
| **11** | **Contingency strategies to ensure food provision remained as close to the original protocol.**  *Researchers performed quality control checks by placing/confirming grocery orders with participants, keeping food stock on hand of essential menu items for participants to collect if required, use of a pre-developed food library/substitutes food list for out-of-stock items* | Brief description (couple of sentences) | 4-6 |
|  |  | Detailed description in supplementary file | Not reported |
| **DOMAIN 3 – DIETARY ASSESSMENT** | | | |
| **12** | **Dietary assessment method(s) used (strengths, limitations, reliability and validity, including whether it has been validated in the population being studied) or reason(s) why a dietary assessment method was not used.**   - *Stating the name of tools, whether it was validated and in what population including references (where relevant).* - *Stating if calibrated against weighed food records (E.g.,* [ASA-24®,](https://epi.grants.cancer.gov/asa24/) [Intake-24](https://intake24.co.uk/)*) and/or validated using strategies such as direct observation, an objective measure (e.g., doubly labelled water), recovery biomarkers, etc.* - *Stating whether participants were asked to return all uneaten food, whether this was weighed/recorded against food provided, etc* - *Stating whether all food was eaten at research facility under supervision* | Brief description (couple of sentences) including statement on validation and relevant references | 6 |
| **13** | **a) Description of the dietary assessment method(s) used to examine food items recorded (or consumed) and estimate (or quantify) portion size.**   - *Serves of each food group, grams of each food or food group via 24-hr recalls etc* - *If validated, reference the validation paper relating to the method/tool* | Brief description (couple of sentences) | 6 |
|  |  | Detailed description in supplementary file and/or example of method/tool used if applicable | N/A |
| **14** | **b) Description of the frequency of conducting the dietary assessment method(s), including number of days (if applicable).**  *Serial 24-hr recalls 4 times per study period, or two 3-day food records at baseline and post-intervention, FFQs, weighed food records weekly, direct meal observation etc* | Described in one sentence or very briefly | 6 |
| **15** | **c) Description of the timing of the dietary assessment method(s) used in relation to the timing of biospecimen data collection.**  *Dietary intake collected 24hrs prior to blood collection, or dietary intake collected at time of biospecimen (urine, blood, faecal, saliva) sample collection* | Described in one sentence or very briefly | 5 – Table 2 |
|  |  | **Optional:** report in a figure | Not reported |
| **16** | **d) Description of how the dietary assessment method(s) were administered and by whom.**  *Interviewer administered (study investigators) or self-administered (participant via e-form, survey etc)* | Described in one sentence or very briefly | 6 |
| **17** | **e) Description of how the quality and accuracy of the administration of the dietary assessment method(s) was assured.**  *Quality control checks e.g., results reviewed by study investigators and clarified with participant where relevant, random phone call audits etc* | Described in one sentence or very briefly | 5-6 |
| **18** | **Qualitative and quantitative dietary intake data for all dietary intervention(s) and control diet(s) and whether data presented is for reported intake or based on foods/beverages provided/prescribed only.**   - *Tabulated servings of foods by food groups for each feeding arm (and whether this is reflective of provision/prescription, reported intake, or both).* - *Tabulated nutritional information for each feeding arm (and whether this is reflective of provision/prescription, reported intake, or both).* - *Incorporating deviations to dietary protocol, either incorporated as part of dietary assessment method (for actual intake reporting) or retrofitted/overlaid on dietary protocol (for intake presented as food provided).* | Detailed description (up to ~250 words) | N/A – protocol paper |
|  |  | Detailed tabulation for each diet group | N/A – protocol paper |
|  |  | Detailed description in supplementary table(s) and/or figure(s) | N/A – protocol paper |
| **19** | **Methods used to assess and account for consumption of non-study food and/or beverage items i.e., foods that were consumed but not provided or prescribed as part of diet protocol.**   - *Log of non-study food/beverage items consumption documented in an online or paper-based proforma list* - *Captured in dietary assessment method* | Brief description (couple of sentences) | 4 |
|  |  | Detailed description in supplementary file | Not reported |
| **20** | **Procedure used to match food composition of dietary intervention items provided with actual consumption data, reporting conversion factors or assumptions made (if applicable).**   - *Food composition databases e.g., Australian Food Composition Database (formerly NUTTAB) used to analyse nutrient intake.* - *Sensitivity analysis to adjust for prescribed v actual dietary intake* | Brief description (couple of sentences) | N/A – protocol paper |
| **DOMAIN 4 – ADHERENCE AND COMPLIANCE MONITORING** | | | |
| **21** | **Method(s), tools, and/or resources used to optimise engagement and adherence to diet intervention(s), and whether this was the same for all diet interventions (where applicable).**   - *Energy-matched / tailoring to food preferences (where possible) and how e.g., unit foods* - *Itemised meal plan with portion sizes* - *Non-study food consumption guide e.g., takeout* - *Provide a meal box/lunch box to support out-of-home consumption* - *Meal box reminder cards of what to pack* - *Check-in phone calls* - *Variability in repeated menus to prevent fatigue (where applicable to research question)* - *Rotating menu with cycle length that prevents fatigue e.g., 7day* - *Reminders e.g., automated email reminders/texts or phone calls.* - *Examining satiety (VAS) and/or food acceptability questionnaire* - *Consultation with research team e.g., email, phone, study interval check-in appts/communication* | Brief description (couple of sentences) | 5-6 |
|  |  | Detailed description in supplementary table(s), figure(s) and/or include examples | Not reported |
| **22** | **Method(s) used to monitor adherence to dietary intervention(s), stating whether this involved objective methods (e.g., biomarkers or known metabolites), and whether the method(s) used was the same for all dietary interventions (where applicable) and control diet(s).**   - *Use of ‘marker foods’ with known metabolites that are measured in biospecimen.* - *Objective measures such as PABA to examine sample collection completeness* - *Where biospecimens are used, state type of biospecimen e.g., plasma, urine, and the nature of collection e.g., spot urine, 24hr collection etc.* - *Dietary assessment methods e.g., 24hr recalls, food records/diaries, direct meal observation.* - *Weighing of uneaten portions and/or uneaten food (including spilled food) returned or photographed* - *Specific compliance questionnaire and/or checklist* - *Full (or at least partial) diet provision* - *Supporting resources e.g., itemised meal plan, meal box reminders, takeout meal ideas* - *Check-in phone calls / regular consultation with researchers* | Detailed description (up to ~250 words). | 4-6 |
|  |  | **Consider:** detailed description in supplementary file | Not reported |
| **23** | **How non-adherence and/or outliers were managed.**   - *Consumption of non-prescribed food, non-consumption of prescribed foods, describe cut-offs that identify non-adherence.* - *Describe procedures that identified outliers to the dietary protocol e.g., excessive metabolite concentrations that can’t be reasonably explained. Include description of cut-offs.* | Brief description (couple of sentences) | 4 (at run-in) |
|  |  | **Consider:** detailed description in supplementary file | Not reported |
| **24** | **Detailed description of how unforeseen circumstances (e.g., acute illness, personal circumstances) that required deviation or adjustment to dietary protocol were managed (e.g., temporary pause in dietary intervention with recommencement after a suitable washout period, adjustments in nutritional requirements, or rescheduling of clinic appointments).**   - *Temporarily pause feeding intervention periods and/or reschedule clinic appointments with a suitable washout period for recovery of illness* - *Ceasing dietary intervention followed by suitable washout period before recommencing dietary intervention* - *Adjustment in nutritional requirements (if relevant)* | Brief description (couple of sentences) | 5-6, 8 |
|  |  | Detailed description in supplementary file | Not reported |
| **DOMAIN 5 – BIAS** | | | |
| **25** | **How selection bias in dietary intervention allocation were mitigated or addressed.**   - *Randomised order of dietary intervention (cross-over study) or allocation to dietary intervention (parallel study).* - *Stratified random sampling (individuals stratified for sex and any other characteristics known to influence the dietary metabolome and/or other key outcomes).* - *If and how blinding was implemented e.g., single, double etc* | Brief description (couple of sentences) | 3 |
| **26** | **Whether a washout period was employed, and if so, what the conditions were, and duration justified.**  *Washout period between dietary interventions such as return to habitual dietary intake or standardised feeding protocol.* | Brief description (couple of sentences) | 1, 3-5 & Table 2 |
|  |  | **Consider**: description in a figure | N/A |
| **27** | **How potential bias in dietary reporting (i.e., misreporting, recall bias, changing habits as a result of being assessed) were mitigated.**   - *Use of validated dietary assessment methods with visual aids to support accurate recall e.g., ASA-24®, Intake-24, Australian Eating Survey (AES)* - *Use of image-based and/or sensor-based dietary assessment methods* - *Interviewer-administered dietary assessment methods* - *Strategies to control for over- and under-reporting e.g., Goldberg equation* | Brief description (couple of sentences) | 4, 6 |
|  |  | **Consider:** detailed description in supplementary file | Not reported |
| **28** | **Measures taken to control for potential confounding factors that could influence inter- and intra-individual variations outside the scope of the study protocol.**   - *Cross-over study design so that participants serve as their own controls.* - *Cross-over study design in random order so that there is no order effect.* - *Provide a standardised dietary run-in phase (e.g., 1-2 weeks) prior to randomisation e.g., whole diet feeding, partial diet feeding, highly prescriptive meal plan.* - *In a parallel study design, standardised test meals or foods administered at various time points throughout the study. These meals/foods would be provided before concurrently testing metabolomic or other metabolic measures to evaluate individual responses.* - *Provision of partial or whole diet to reduce variability in food preparation or cooking practices.* | Brief description (couple of sentences) | 1, 3-5 |
| **29** | **Acknowledgement of the generalisability of the population being studied.**  *Comment on the generalisability of population being studied.* | Described in one sentence or very briefly | Not reported |

^†^ Provide details of where this information is available/sourced from if it is not provided in the current paper. For example, citations for published papers or protocol papers, website URL, and/or catalogue or report citations. Describe any derivations or deviations from original protocol. We strongly recommend using this checklist in conjunction with the DID-METAB Explanation and Elaboration report (*add citation and DOI)* which provides further information.

**Supplementary File References**

1. Asher RC, Jakstas T, Lavelle F, Wolfson JA, Rose A, Bucher T, et al. Development of the Cook-Ed(TM) Matrix to Guide Food and Cooking Skill Selection in Culinary Education Programs That Target Diet Quality and Health. Nutrients. 2022;14(9).

2. Niederberger M, Spranger J. Delphi Technique in Health Sciences: A Map. Front Public Health. 2020;8.

3. Garcia-Perez I, Posma JM, Gibson R, Chambers ES, Hansen TH, Vestergaard H, et al. Objective assessment of dietary patterns by use of metabolic phenotyping: a randomised, controlled, crossover trial. Lancet Diabetes Endocrinol. 2017;5(3):184-95.

4. Gürdeniz G, Uusitupa M, Hermansen K, Savolainen MJ, Schwab U, Kolehmainen M, et al. Analysis of the SYSDIET Healthy Nordic Diet randomized trial based on metabolic profiling reveal beneficial effects on glucose metabolism and blood lipids. Clin Nutr. 2022;41(2):441-51.

5. Ferguson JJA, Clarke E, Stanford J, Burrows T, Wood L, Collins C. Dietary metabolome profiles of a Healthy Australian Diet and a Typical Australian Diet: protocol for a randomised cross-over feeding study in Australian adults. BMJ Open. 2023;13(7):e073658.
